# Study protocol on Enhanced Primary Healthcare (EnPHC) interventions: a quasi-experimental controlled study on diabetes and hypertension management in primary healthcare clinics

**DOI:** 10.1017/S1463423620000250

**Published:** 2020-08-13

**Authors:** Sheamini Sivasampu, Xin Rou Teh, Yvonne Mei Fong Lim, Su Miin Ong, Swee Hung Ang, Masliyana Husin, Noraziani Khamis, Faeiz Syezri Adzmin Jaafar, Wen Jun Wong, Sunita Shanmugam, Siti Aminah Ismail, Sarah Hui Li Pang, Nazrila Hairizan Nasir, Mohd Safiee Ismail, Dian Kusuma, Dennis Ross-Degnan, Rifat Atun

**Affiliations:** 1 Institute for Clinical Research, National Institutes of Health, Ministry of Health Malaysia, Selangor, Malaysia; 2 Institute for Health Management, National Institutes of Health, Ministry of Health Malaysia, Selangor, Malaysia; 3 Family Health Development Division, Ministry of Health Malaysia, Putrajaya, Malaysia; 4 Harvard T.H. Chan School of Public Health, Boston, MA, USA; 5 Harvard Medical School, Harvard University, Boston, MA, USA; 6 Harvard Pilgrim Health Care Institute, Boston, MA, USA

**Keywords:** EnPHC, hypertension, person centered, primary healthcare, study protocol, Type 2 diabetes mellitus

## Abstract

**Aim:**

This paper describes the study protocol, which aims to evaluate the effectiveness of a multifaceted intervention package called ‘Enhanced Primary Healthcare’ (EnPHC) on the process of care and intermediate clinical outcomes among patients with Type 2 diabetes mellitus (T2DM) and hypertension. Other outcome measures include patients’ experience and healthcare providers’ job satisfaction.

**Background:**

In 2014, almost two-thirds of Malaysia’s adult population aged 18 years or older had T2DM, hypertension or hypercholesterolaemia. An analysis of health system performance from 2016 to 2018 revealed that the control and management of diabetes and hypertension in Malaysia was suboptimal with almost half of the patients not diagnosed and just one-quarter of patients with diabetes appropriately treated. EnPHC framework aims to improve diagnosis and effective management of T2DM, hypertension or hypercholesterolaemia and their risk factors by increasing prevention, optimising management and improving surveillance of diagnosed patients.

**Methods:**

This is a quasi-experimental controlled study which involves 20 intervention and 20 control clinics in two different states in Malaysia, namely Johor and Selangor. The clinics in the two states were matched and randomly allocated to ‘intervention’ and ‘control’ arms. The EnPHC framework targets different levels from community to primary healthcare clinics and integrated referral networks.

Data are collected via a retrospective chart review (RCR), patient exit survey, healthcare provider survey and an intervention checklist. The data collected are entered into tablet computers which have installed in them an offline survey application. Interrupted time series and difference-in-differences (DiD) analyses will be conducted to report outcomes.

## Introduction

The burden of non-communicable diseases (NCDs) such as cardiovascular diseases (CVDs), chronic obstructive lung diseases, cancer and diabetes affects health and the socio-economic development of countries (Mendis, 2014; Bommer *et al*., 2018; Niessen *et al*., 2018). High systolic blood pressure, fasting plasma glucose and body mass index are the leading risk factors of disability-adjusted life years globally (GBD 2017 Risk Factor Collaborators, 2018). These metabolic risk factors are known to increase CVD which is the main contributor to NCD-related premature death (World Health Organization, 2018). However, the causes of CVDs are largely amenable to change through behavioural modification and risk factors prevention.

A comprehensive situational analysis of the Malaysian health system undertaken in 2016–2018 revealed that the NCD burden is very high and is rapidly increasing with almost two-thirds of Malaysian adult population having at least one of the three NCDs – Type 2 diabetes mellitus (T2DM), hypertension or hypercholesterolaemia (Institute for Public Health, 2015; Ministry of Health Malaysia and Harvard University TH Chan School of Public Health, 2016). This analysis, which used a ‘care cascade’ framework, revealed major gaps in awareness of risk, timely screening, definitive diagnosis, engagement and treatment for T2DM, hypertension and hypercholesterolaemia (Institute for Public Health, 2015). The shortcomings in NCD care in primary healthcare clinics are mostly due to lack of continuity and coordination of care, poor organisational management of healthcare providers, long waiting times and limited operational hours, low screening and counselling activities, limited awareness of the need for screening services and preventive care and suboptimal therapeutic prescribing for the treatment of T2DM and hypertension (Ministry of Health Malaysia and Harvard University TH Chan School of Public Health, 2016). These findings reflect the need to address the care gaps in the health system to effectively prevent and treat CVDs.

In order to improve the performance of the Malaysian health system in managing NCDs, an integrated care model was designed to enhance existing healthcare services by incorporating public health, primary healthcare and social support as part of a ‘network’ linked to appropriate secondary and tertiary hospital services. This new framework, called ‘Enhanced Primary Healthcare’ (EnPHC), uses primary healthcare as an agent of change to deliver efficient and effective service and in turn contributes to the entire care continuum. EnPHC framework includes multiple interventions: (i) establishment of a population database with population enrolment and risk profiling, (ii) community-based intervention programmes, (iii) branding and social marketing, (iv) development of integrated multidisciplinary care pathways based on international evidence with local adaptation, (v) continuous improvement of care delivery, (vi) improved organisational practices and (vii) integrated care networks (Institute for Public Health, 2019b). All interventions are necessary for the cohesive and successful implementation of EnPHC but adaptations to the intervention are allowed.

EnPHC aims to improve management of T2DM, hypertension and hypercholesterolaemia across the cascade of care by increasing early detection and management of the target NCDs, surveillance and monitoring of diagnosed patients with improved continuity and coordination and by providing enhanced person-centred quality of care. The EnPHC framework is implemented as a demonstration project to inform decisions on further expansion to the rest of the country (Institute for Public Health, 2019b). The implementation is carried out by the Family Health Development Division of Ministry of Health Malaysia, and the evaluation is a collaborative effort between Institute for Clinical Research (ICR), Institute for Health Management (IHM), Institute for Public Health (IPH), Institute for Health Systems Research (IHSR) and Institute for Health Behavioural Research (IHBR), working with researchers at Harvard University.

The conceptual framework to guide the implementation and evaluation is discussed below. An impact evaluation that spans from the community to the patients and providers of primary healthcare is carried out (Institute for Public Health, 2019a). To further strengthen the findings from the impact evaluation studies, a process evaluation study is carried out to evaluate the implementation process in terms of fidelity and quality of implementation (Institute for Health Systems Research, 2019).

To evaluate the interventions, complementary data from multiple sources are used to measure outcomes. Evaluation performed at primary healthcare clinics aims to measure health service and patient outcomes. Therefore, the primary objective is to evaluate the impact of EnPHC interventions on processes of care and intermediate clinical outcomes among T2DM and hypertension patients, and the secondary objective is to evaluate the impact of the EnPHC interventions on patients’ experience and providers’ job satisfaction. The aim of this paper is to describe the sequential steps taken to evaluate the EnPHC interventions at primary healthcare clinics (EnPHC-EVA: Facility) and the methods used.

### EnPHC interventions

EnPHC interventions started from July 2017 and took three months for all clinics to fully implement. The EnPHC interventions represent activities in the community, facilities and networks targeting the three NCDs and risk factors (Institute for Public Health, 2019b). The EnPHC intervention is aimed at two levels which are the community and the facility level. The community level targets Malaysian adults aged 18 and above, who live within the operational area of the primary healthcare clinics. The facility level targets Malaysian adults aged 30 and above, utilising the primary healthcare clinics. The seven components of the new EnPHC framework (the intervention) are listed in the introduction and the comparison of the current public sector primary healthcare model in Malaysia, with the EnPHC framework shown in Table 1 (Institute for Public Health, 2019b).

**Table 1.** Components of the EnPHC intervention and comparison of the current public sector primary healthcare model in Malaysia with EnPHC model

Component	Definition	Current model	EnPHC model
**(i) Population health database with population enrolment and risk profiling**			
Population catchment areas	Establishment of population catchment areas for primary healthcare clinics.	Limited availability	Available
Population health database	Development of a national population health database by data linkage from the National Registration Department and Ministry of Health. Data on screening will be entered using the MySihat Online Evaluation System (MOVeS) and fed into the database.	Limited availability	Available
Risk profiling with targeted messaging using Malaysian Non-communicable disease (NCD) risk calculator and NCD risk assessment	Systematic process of profiling behavioural and clinical health risks. Using population data, risk profiles of population groups or individuals can be developed by applying mathematical modelling focusing on hypertension and Type 2 diabetes mellitus (T2DM) and their risks being high cholesterol, obesity, physical inactivity and smoking. These stratification will be fed back to the respective District Health Office for further intervention.	Not available	Available
**(ii) Community-based intervention programme**			
Mobile health teams and community outreach efforts	Community health coordinator (CHC) is introduced at the district level, which acts as a proactive link between the clinics and the community. CHC is a medical officer (MO), who is in charge of the NCD unit in the district health office. CHC will liase with the non-govermental organisations, Healthy Community Empowers the Nation (KOSPEN), other community organisations and mobile health teams from the clinics to coordinate the following activities: (a) Health promotion and prevention (b) Health screening (c) Enrolment outreach (d) Defaulter outreach	Not available	Available
**(iii) Branding and social marketing**			
Strategic communication on health education and services to the community	Information on health education and services will be disseminated via different modes: (a) Social media (b) Ministry of Health website (c) Printed media (d) Others (e.g. local radio and state health promotion unit)	Limited	More extensive
**(iv) Integrated multidisciplinary care**			
Integrated care pathway (ICPs)	A cardiovascular care bundle that consists of ICPs—hypertension and T2DM. Care pathways are a series of management guidelines usually developed in the form of flow charts that guide care providers on how to provide treatment for all patients with a specific condition. These guidelines are designed with patient-centric ‘workflows’ and are utilised by a multidisciplinary team with specific tasks each team member at each critical step can take across the care continuum. Each ICP consists of five process elements: (a) Screening (b) Diagnosis (c) Risk profiling (d) Risk stratification and management (e) Referral	Comprehensive but designed as guidelines	Comprehensive but more operationally feasible
Visit checklist (VC)	VC is a checklist in the form of Microsoft Excel designed help family health teams (FHTs) standardise practices, improve efficiency and optimise patients’ visits. It is able to function as a ‘to do’ list in preparation for a patient’s appointment and capture current patient information such as key vital signs, lab results and medications for each visit. This will create a database on the ongoing services provided to patients.	Not available	Available through provider apps or paper-based forms to promote standardisation of care provision
Integrated specialised services (ISS)	ISS are the allied health members who are specifically trained for detailed management of cases in their area of specialisation. This includes an occupational therapist, physiotherapist, social workers, pharmacist, NCD educator, dietician and nutritionist. This would allow the MO to refer to such services and have more time to manage moderate or poorly controlled patients.	Limited availability	Available (visiting basis in most clinics)
Cardiovascular Care Bundle Medication Therapy Adherence Clinic (CCBMTAC)	CCBMTAC will be carried out by the pharmacists to assess patient compliance, conduct medication review, direct intervetion or referral to FHT and provide recommendation for those patients who have low medication adherence. Medication adherence will be assessed via both objective (medication possession ratio) and subjective (interview) measures.	Limited Medication Therapy Adherence Clinic (MTAC) service and disease specific (diabetes)	CCBMTAC covers the care bundle with structured feedback to FHT
Improve information systems for providers	A single electronic information system that integrates existing systems, provides options for manual and digital entry and supports the health system as it moves towards its long-term Tele-Primary Care (TPC) transformation.	Paper-based clinical records, legacy TPC systems and TPC-OHCIS	Paper-based clinical records, legacy TPC, TPC-OHCIS v 1.1, mobile patient and provider apps
**(v) Continuous improvement of care delivery**			
Clinical and prescribing audits	Clinical and prescribing audits are intended as routine assessments of care. Details in the indicators are available here (46).	Annual audit (e.g. National Diabetes Registry audit)	Additional indicators are measured and monthly or quarterly performance reports are generated
Secondary triage (screening and cardiovascular risk stratification)	Enhancements such as increasing the screening coverage for all the adults ≥ 30 years old; usage of NCD screening form and cardiovascular risk stratification using Framingham risk score are performed at the secondary triage. After being risk stratified, low-risk patients will be managed by assistant medical officer (AMO)/nurse, whereas medium- and high-risk patients will be managed by MO.	Screening counter is available	Available
**(vi) Improved organisational practices**			
Primary Health Team (PHT) and FHTs	Two or more FHTs are formed within a single PHT for each clinic. Each FHT will function as a multidisciplinary unit that provides services for all patients within a zone in a catchment area. At a minimum, each FHT core team will include a family medicine specialist (FMS), a MO, an AMO/nurse and a care coordinator (CC). FMSs and CCs will work on multiple FHTs within a clinic, ensuring that care across FHTs is uniform. FHT will not just assess, diagnose and manage its patients but will be responsible for educating, coordinating referral and maintaining good record keeping of each assigned zone.	Physician-centred care with limited roll out of family doctor concept (FDC)	FHTs
Primary triage and standardising signage at clinics	A primary triage and improved standardised signages are introduced to decongest the registration counter and ensure smooth navigation for patients as they go through the check points for a visit within the clinic.	Limited availability	Available
CC	CCs are essential for creating and maintaining a strong link between patients and their FHT providers. CCs will work closely with community workers, providers at screening centres and secondary and tertiary care centres to keep FHTs informed about the status of patients and any population changes in catchment areas. They are also responsible for connecting patients with support services and managing referrals and counter-referrals. CC will need a qualification of paramedic (either AMO or nurse) who has been working with the Ministry of Health and received certified training in primary healthcare or NCD management or senior paramedics with five days training in NCD management, ICP, communication and management.	Not available	Available
NCD care form	NCD Care form is a monitoring tool that includes patient health information, treatment and referral status for a specific NCD or a group of NCDs. Clinical teams will use this tool to continually assess the needs of and care provided to patients. FHTs will routinely review the forms to identify gaps in patient care and analyse data to identify where the quality of patient care may be improved. During Clinical and Prescribing Audits, data assembled will be used to study clinic performance and provide ongoing feedback about team effectiveness. NCD Care form will be used as a standardised referral document for referral to secondary/tertiary care and also referral to ISS.	Diabetic book, NCD book (Unstandardised)	Available for multiple NCDs for FHT use
**(vii) Continuity of care across healthcare facilities and communities**			
Referral and counter-referral	For advanced cases that require treatment beyond clinic capacity, patients with NCD will be referred to secondary or tertiary care. CCs will track patient referrals and counter-referrals and will continue to monitor patients along the continuum	Available but poor efficiency	Implemented by CCs
NCD Care Form	NCD Care Form will be used as the standard referral document to ensure all necessary information is conveyed during the referral process.	Referral form available but insufficient information captured	NCD Care Form with patient history and management plan

EnPHC, enhanced primary healthcare.

Note: Adapted from the Malaysia Health Systems Research Study: Implementation Plan 2016 and Enhanced Primary Healthcare Lab 2017.

### Conceptual framework

**Figure 1.**
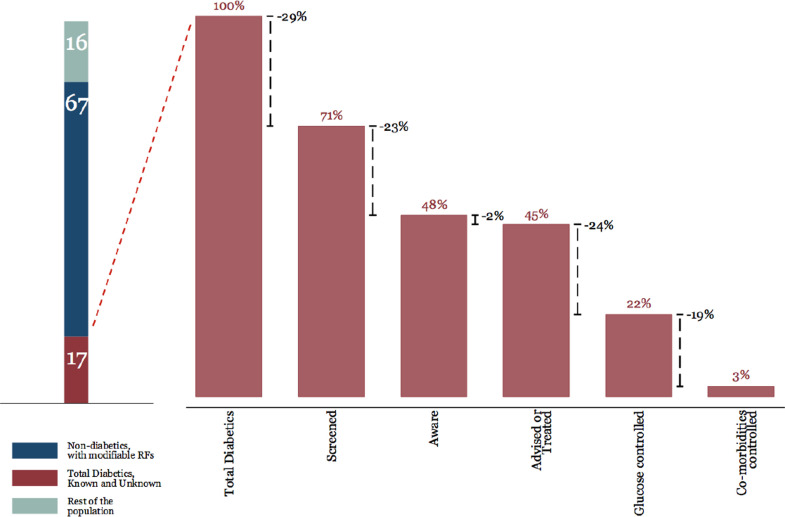
Care cascade for T2DM in Malaysia (Atun *et al*., 2016).

We used a health systems approach when conceptualising multiple interventions along the care cascade – as improvements in outcomes will require addressing every step in the care cascade. Care cascades have been used to examine the effectiveness and efficiency of management of infectious diseases in health systems, for example, for human immunodeficiency virus (HIV) (Gardner *et al*., 2011), Hepatitis C (Yehia *et al*., 2014), tuberculosis (Kim *et al*., 2019), as well as for NCDs such as diabetes (Prenissl *et al*., 2019a) and hypertension (Prenissl *et al*., 2019b) and examine health system performance using these conditions as tracers (Manne-Goehler *et al*., 2016; Manne-Goehler *et al*., 2019). In essence, the Malaysian care cascades for T2DM and hypertension refer to four main steps, which include proportions of patients who are (i) screened, (ii) aware of their disease, (iii) treated or advised and (iv) have their disease under control (Prenissl *et al*., 2019a; 2019b). Figure 1 shows the Malaysian T2DM care cascade which revealed that there is under-detection and under-treatment (Atun *et al*., 2016).

Our theory of change is that improvements in every step of the care cascade are necessary if outcomes are to improve, and the use of evidence-based integrated care pathways (ICPs), supported by the use of clinical audits (Grimshaw *et al*., 2001) and ongoing learning (Ivers *et al*., 2012) that optimised efficiency and effectiveness of services provided along the care cascade, would help improve health outcomes, user experience and user satisfaction (Baxter *et al*., 2018).

## Methods

### Study design and study sites

The EnPHC-EVA: Facility study is a quasi-experimental controlled study which involves 20 intervention and 20 control clinics. We drew on a methodology developed by Imai, King and Nall (Imai *et al*., 2009). After considering regional representativeness, budget and capacity to implement EnPHC interventions, 20 matched pairs from two states (Johor and Selangor) were selected to be the study sites. The clinics were matched based on the criteria listed in Table 2 (Family Health Development Division, 2018). In addition, ArcGIS software (ESRI, 2011) was used to ensure that the matched pairs were in different districts to minimise patients’ overlap. The matched pairs were then randomly allocated to the ‘intervention’ or ‘control’ arm of the study by ‘flipping a coin’.

**Table 2.** Clinic inclusion and matching criteria

Clinic inclusion criteria	Clinic matching criteria
Has at least two medical officers Clinics with attendances between 300 and 800 patients per day	Number of medical officers Number of Family Medicine Specialist Location: Rural/Urban Availability of Tele-Primary Care system Annual patient attendance

**Figure 2.**
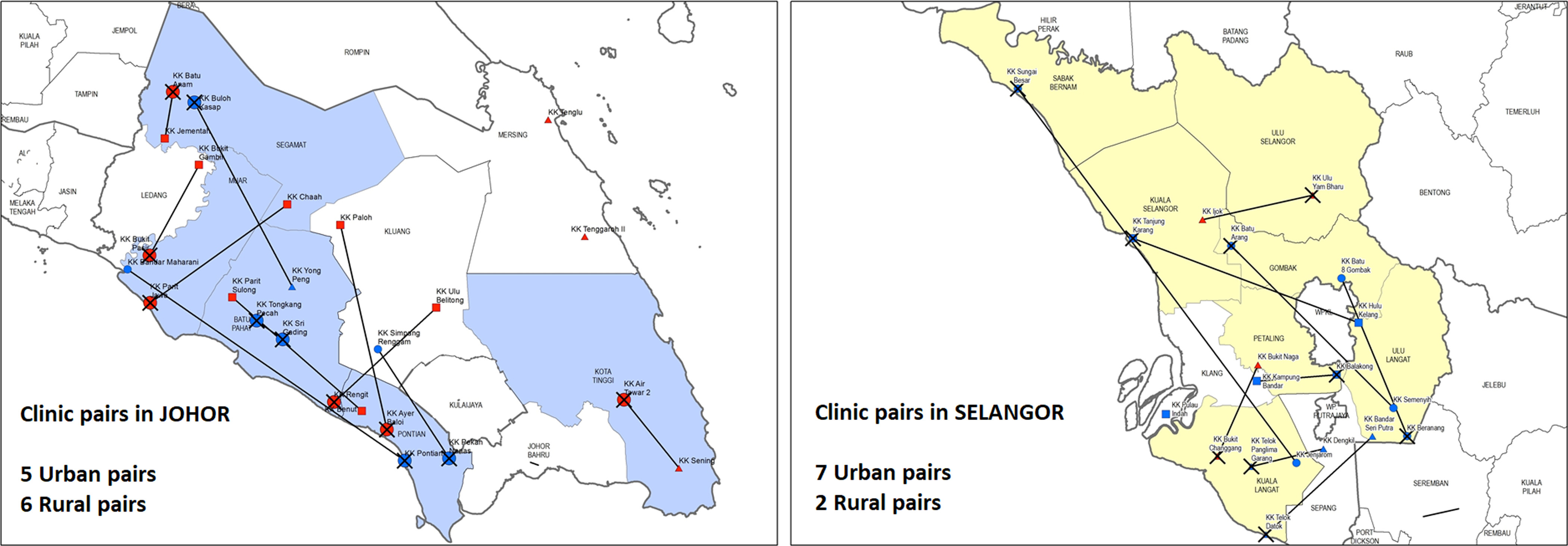
Selected clinic pairs in Johor and Selangor.

There are nine and 11 matched pairs, in Selangor and Johor states, respectively. Figure 2 shows the matching pairs in both Johor and Selangor states, where intervention clinics were identified with crosses.

### Study population

The criteria for selecting the sample for each questionnaire are listed in Table 3. The different questionnaires used are described under the subheading *Data Collection Tools*. We include patients of 30 years and above to match with EnPHC interventions. Pregnant women are not included as they would have different clinical management plans.

**Table 3.** Inclusion and exclusion criteria for retrospective chart review, patient exit survey and provider survey

	Inclusion Criteria	Exclusion Criteria
Retrospective chart review (RCR)	Malaysian Aged 30 years and above Had documented diagnosis of Type 2 diabetes mellitus (T2DM) or hypertension	Pregnant
Patient exit survey	Malaysian Aged 30 years and above Had documented diagnosis of T2DM and/or hypertension Had clinic visit for T2DM and/or hypertension on the day of survey	Pregnant
Provider survey	All permanent and visiting providers on duty in the clinic during the data collection period	Providers who served less than a month in that clinic

### Outcome measures

Outcome measures used in this study are captured using different data collection tools and are summarised in Table 4. It is anticipated that improvements of blood pressure, lipid levels and glycaemic control will be observable after at least one year as changes have been detected after 12.5 months of implementation in similar evaluation studies (Norris *et al*., 2001). Hence, for post-intervention, information on processes of care (investigations, counselling and prescribing patterns) will be gathered via RCRs 15 months after implementation.

**Table 4.** Outcome measures of the EnPHC-EVA: facility study

Outcomes	Indicators		Data Source
	T2DM	Hypertension	
Process of care	Proportion of Patients whose BP and glucose were measured at every visit Patients with HbA1c tests done at least once over the past three months Patients with weight and BMI assessed at least once over the past six months Patients with the following examinations done at least once a year: lipid profile tests, serum creatinine, blood urea nitrogen and urine albumin tests, eye examination (visual acuity and fundus), foot examination and liver function test Patients with cardiovascular risk assessment done within the past one year Patients who received counselling: exercise, dietary Patients who received pharmacotherapy: lipid lowering drugs, insulin and antihypertensives	Proportion of Patients with BP measured at every visit Patients with fasting blood glucose, serum creatinine, urine albumin and lipid profile measured at least once a year Patients with electrocardiogram and cardiovascular risk assessment done within the past one year Patients who received counselling: exercise, dietary Patients who received pharmacotherapy: antihypertensives	RCR
Intermediate clinical outcomes	Proportion of Patients with HbA1c ≤ 7% Patients with BP ≤ 135/75 mmHg Patients with LDL-C ≤ 2.6 mmol/L Patients with HDL-C > 1.0 mmol/L (male) or HDL-C > 1.2 mmol/L (female)	Proportion of Patients with BP < 140/90 mmHg Patients with LDL-C ≤ 2.6 mmol/L Patients with HDL-C > 1.0 mmol/L (male) or HDL-C > 1.2 mmol/L (female)	RCR
Patient disease awareness	Proportion of patients who know their diagnosis		Patient exit survey
Patient experience	Mean score in assessment of care for chronic illness (activation, decision support, goal setting) Proportion of patients who perceived effective patient–provider communication Proportion of patients whose waiting and consultation time matched their expectation		Patient exit survey
Patient satisfaction	Proportion of patients who are willing to recommend the clinic to friends and family		Patient exit survey
Providers’ satisfaction	Mean score on six components of job satisfaction among providers (stress, administrative work, interest, balance between effort and reward, respect and whether their job makes sense to them)		Provider Survey
Providers’ quality of care	Proportion of providers providing quality care and continuous care		Provider survey

BP: Blood pressure; HbA1c: Haemoglobin A1c; BMI: Body mass index; LDL-C: Low-density Lipoprotein Cholesterol; HDL-C: High-density Lipoprotein Cholesterol; TG: Triglyceride.

Improved self-efficacy, improved health beliefs and a clear care pathway are expected to increase patients’ satisfaction with the healthcare system and improve utilisation (Panagioti *et al*., 2014). These outcomes will be captured via a patient exit survey. In addition, this study also includes a measure of potential unintended outcomes, such as the job satisfaction of the healthcare providers involved in implementing the interventions. Patient exit survey and provider survey will be carried out at 20 months post-intervention. In the long run, there are expected to be reductions in complications of T2DM, hypertension and hypercholesterolaemia and improvement in patients’ quality of life. However, this evaluation study was not designed to measure the changes in hypercholesterolaemia and the long-term outcomes.

### Data collection tools and sources

This study gathers data from patient medical records, a self-administered questionnaire for healthcare providers and patient interviews. The data collection tools include a data extraction form for RCR, three questionnaires (patient exit, provider and facility questionnaires) and an intervention checklist. All data collection tools are available in Appendices A–E. All the data collection tools underwent a pre-test for comprehensibility.

#### Data extraction form for retrospective chart review

An electronic data extraction form was designed to collect information on the process of care and intermediate clinical outcomes, as described in Table 4. The source of data extraction is T2DM and hypertension patients’ medical records that were available as paper-based documents, within the legacy tele-primary care (TPC) computer system or any standalone electronic medical system.

#### Patient exit questionnaire

The research committee comprising intervention implementers, family medicine specialists (FMS), public health specialists and study researchers had reached a consensus to adapt a subset of items from the Quality and Cost of Primary Care (QUALICO-PC): Patient Experience Survey (Sivasampu *et al*., 2016) and the Patient Assessment of Chronic Illness Care (PACIC) survey instruments (Glasgow *et al*., 2005) for the patient exit questionnaire. This decision was reached after taking into account the length of the questionnaire and health literacy level of the study population (Azreena *et al*., 2016) in order to reduce respondent fatigue and increase completion rates. The questionnaire contained 43 items and was completed through face-to-face interviews, which measure socio-demographic characteristics, patient experience and satisfaction, disease awareness and self-management support.

#### Provider questionnaire

Similarly, the research committee adapted the questions from QUALICO-PC: General Practitioner questionnaire (Sivasampu *et al*., 2016) for the provider questionnaire. The main aim of the provider survey is to gather information on job satisfaction. The decision to measure job satisfaction is based on the conceptual framework that was described in the earlier section. Six components of job satisfaction are measured, which include stress, administrative work, interest, balance between effort and reward, respect and whether their job makes sense to them. It also measures the use of latest clinical practice guidelines, continuity and coordination of care and workload. As such, a self-administered provider questionnaire consisting of 28 questions, which focused on the structural aspects of primary care, workload, demographic and the provider’s satisfaction, was developed.

#### Facility questionnaire

The facility questionnaire was developed to measure information on clinic resources, including staff, infrastructure and equipment, as well as services provided, which can determine the success of the intervention.

#### Translation of English version questionnaires into Malay language

The translation of the English version of the patient exit questionnaire, provider questionnaire and facility questionnaire into Malay language was performed using forward and backward procedures. Two study collaborators who were fluent in both English and Malay and familiar with the primary care practice prepared the Malay questionnaire. The Malay questionnaire was then back-translated into the English version independently by another two translators. The researchers compared both versions to ensure that no change in the context occurred during translation and discrepancies were resolved prior to pre-test of the surveys. Subsequently, modifications of the Malay version questionnaire were made in accordance to findings of the pre-test.

#### Intervention checklist

As the EnPHC framework is a complex intervention with multiple elements, it is important to measure the details of its implementation at each clinic in order to quantify the percentage of the intended interventions that actually took place, the level of contamination within control clinics and also to determine the correlation between the degree of implementation and the observed outcomes (O’Donnell, 2008). This is important as contamination is expected as some of the EnPHC intervention components are built upon existing primary care programmes (Safurah *et al*., 2018) (Refer to Table 1).

The 18-item intervention checklist was checked for content validity through a consensus meeting with ten content experts who were EnPHC intervention implementers. The implementers weighed the items based on the expected impact of each item on the study outcomes. The final score of the intervention checklist will be reported as a percentage and used as a covariate in the analysis.

### Study procedures

#### Preparation

A pre-test was carried out in two primary healthcare clinics that were not in the study sample to confirm the strengths and weaknesses of the developed questionnaires. Patients of differing ages, genders, education levels and languages were interviewed and comments on the interview and questionnaire were recorded. Similar steps were applied to the provider questionnaire. Following that, all necessary changes and improvements to the questionnaire were made. Also, the study team did a trial run of the processes for data collection such as tracing patient medical records and interviewing patients and providers. The duration taken for each of these processes was also recorded. This allowed for further refinement of the data collection process and estimation of missing medical records, which can be accounted for in the sample size calculation.

This study recruits fieldworkers who are either medical or pharmacy graduates as data collection requires fieldworkers to be familiar with extracting data from medical records. Fieldworker training included the conduct of quantitative data collection such as manual data extraction from paper-based records, patient recruitment procedures and interview techniques.

#### Data collection period

**Figure 3.**
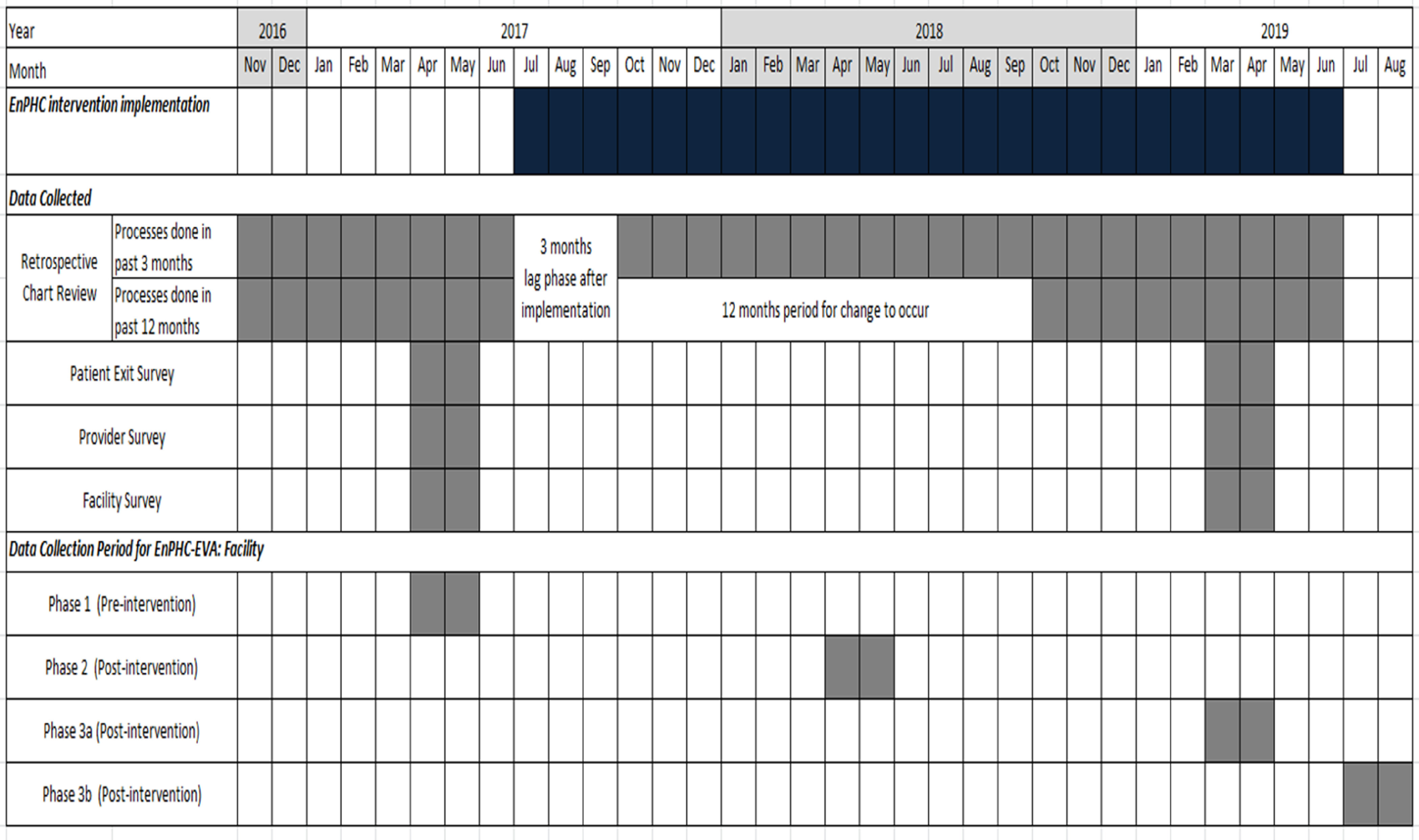
Study timeline and data collection period for EnPHC-EVA: Facility.

The availability of documents and number of fieldworkers were taken into consideration when planning the data collection processes. Data for RCR will be collected for 31 time points (i.e. in months): (i) from November 2016 to June 2017 (pre-intervention) and (ii) from August 2017 to June 2019 (post-intervention). To keep the workload manageable and prevent exhaustion among the study team members, data collection for the 31 time points was divided into four phases, one pre-intervention phase and three post-intervention phases. Data collection phases were also decided based on the feasibility of recruiting for patient interviews during the fasting month. The data collection period for each phase is indicated in Figure 3. Each team is led by one team leader and four fieldworkers.

#### Quality control

Validation rules were embedded within the mobile applications to limit data entry error. The information captured will be uploaded to a central database with continuous monitoring by a data management team that performs quality checks and addresses discrepancies. The final captured data will undergo a systematic data cleaning process preceding data analysis. There is a full audit trail for data management which covers data entry and any amendments to the data. This measure will ensure that all the changes are monitored and there will be no accidental deletion.

### Target sample size and sampling

#### Retrospective chart review

Data analysis for RCR will use two methods – DiD analysis and interrupted time series (ITS) analysis. For DiD analysis, the sample size was calculated using *clustersampsi* in STATA version 14.2 (StataCorp, 2015).

First for T2DM, 1800 patient visits (900 in each arm) were needed to have 80% power to detect a relative change of 28% in the proportion of the patients receiving an annual HbA1c test from the baseline proportion of 52.5% (Mastura *et al*., 2011). This sample size was calculated while taking into account the estimated cluster effect [intracluster correlation (ICC) = 0.091] (Singh *et al*., 2015; Solorio *et al*., 2015). The minimum sample size was rounded up to 2000 or a cluster size of 50 patient visits per clinic. Therefore, the final target sample size for T2DM was 84 patient visits per clinic, after adjusting for an estimated 40% untraceable records.

Second, for hypertension, 1760 patient visits (880 in each arm) were needed to have 80% power to detect a 28% relative change in the proportion of hypertension patients with at least one lipid profile from the baseline proportion of 46% (Wong *et al*., 2015). This calculation took into account the estimated clustering of this outcome at the clinic level (ICC = 0.076) (Singh *et al*., 2015). After adjusting for 40% potentially untraceable records, the sample size required for hypertension patient visits was also 84 patient visits per clinic.

For ITS analysis, a minimum of 100 patient visits per arm at each time point is needed (Wagner *et al*., 2002). A minimum of eight time points was needed for both before and after intervention. In this study, eight time points pre-intervention and nine time points post 15-month intervention were chosen to evaluate the trend and level change over time. For each clinic, the estimated sample size required is ten patient visits for each disease. The final sample size after accounting for 40% untraceable records was 17 patient visits per clinic for each disease.

Systematic random sampling is used to sample the medical records by creating a sampling frame from the patients’ register either in a paper or in an electronic format. In facilities where neither of these is available, the lists are created from patient appointment books.

#### Patient exit survey

For the patient exit survey, we aim to detect a 15% change in the proportion of patients who would recommend the clinic to family or friends, while accounting for an estimated ICC from a previous study of 0.116 (Sivasampu *et al*., 2016). With a statistical power of 80%, we would require at least 920 patient surveys (460 in each arm). Hence, the data collection teams aim to recruit 23 patients per clinic. Patients are sampled consecutively using convenience sampling until the required numbers are achieved.

#### Provider survey

All clinic staff who meet study inclusion criteria will be interviewed. All visiting staff who visit the clinic on a regular schedule are also included in the survey. As EnPHC interventions would involve a change in clinic flow and staff allocation, it is necessary to measure the change in numbers for all categories of staff.

### Data analysis

The data will be analysed using STATA version 14 and R version 3.3.2 (StataCorp, 2015; RStudio Team, 2016; R Core Team, 2017). Descriptive data will be expressed as means or proportions with their corresponding 95% confidence intervals or medians with interquartile ranges. Statistical significance will be determined with a 2-sided *P*-value with *P* < 0.05. A complete case analysis will be used.

An interim analysis will be conducted at the end of Phase 2. A research management team is tasked to monitor the study at the end of Phase 2 and Phase 3b. The research management team will review and comment on the interim and final analysis.

#### Difference-in-differences

DiD analysis will be used to analyse all outcome measures. DiD analysis measures the average effect of the interventions, taking into account two differences: first is the difference between the intervention and control arm and second is the difference between pre- and post-intervention (Gertler *et al*., 2011; Dimick *et al*., 2014). As this is a large-scale field experiment, the difference at baseline is often not negligible and must be accounted for when measuring the intervention effect.

A multivariable model that controls for patient- and clinic-level covariates will be used. Details on the key covariates for each objective can be found in Appendix F. For RCR, patient visits from November 2016 to June 2017 will be grouped to estimate outcome levels in the pre-intervention phase and those from October 2018 until June 2019 will be grouped to estimate outcomes after 15 months of intervention.

Generalised estimating equations will be used to estimate the parameters for both linear and non-linear outcomes while adjusting for clustering at the clinic level. Thus, cluster robust standard errors will be reported. In addition, the key assumptions for DiD analysis which are ‘common shocks’ and ‘parallel trends’ will be checked where trend data are available.

The ‘geeglm’ function from the ‘geepack’ package in *R* will be used to perform the analysis (Yan, 2002; Yan and Fine, 2004; Højsgaard *et al*., 2005). For RCR outcome measures that will be reported as proportions, the DiD estimates will be reported as odds ratios with 95% confidence intervals. For provider survey and patient exit survey outcome measures, the DiD estimates will be reported as changes in means with 95% confidence intervals.

#### Interrupted time series

ITS analysis will examine changes in trends and levels of the outcome measures using monthly data. The observation period started in November 2016 while the interventions were implemented from July 2017. Data from August 2017 to September 2018 will be used to plot a segment to show trend or level change during intervention whereas data from October 2018 to June 2019 will be used to measure the change after 15 months of intervention. ITS analysis can provide an insight into the expected trends of changes, especially for measures that do not show immediate effects. This can show the potential benefits of the interventions, even though the differences in levels are not significant in the early stage of the intervention.

Equivalence of baseline trends in intervention and control groups will be checked. If equivalence cannot be ensured, we will not infer causality between intervention and outcome (Lopez Bernal *et al*., 2018). We assume an impact model with both level and trend change for all outcomes. The final model will be estimated using generalised least squares. All covariates are included based on clinical relevance, hence this will be retained in the model regardless of statistical significance. The ‘nlme’ package in *R* will be used for segmented regression (Pinheiro *et al*., 2013). Results will be presented in ITS charts with the corresponding statistical results for trend and level changes post-intervention.

## Discussion

The EnPHC intervention and evaluation studies aim to apply a set of multifaceted, person-centred interventions that are believed to be effective on the overall management and prevention of NCDs. This is the first study of its kind in the Malaysian context where a public health intervention involving a community outreach component, together with changes at the health system, facility, and patient level, is empirically evaluated in a ‘real-world’ setting. The community outreach component is evaluated by a separate study team and will not be discussed here. The EnPHC intervention was designed to provide organizational-level solutions such as integrated multidisciplinary care, continuous improvement of care delivery and improved organisational practices. It is aimed that, with all intervention components coming together, we will observe from the facility and health providers’ perspective increases in screening and detection rates for NCDs including T2DM, hypertension and hypercholesterolaemia and improvements in processes of care and adherence to T2DM, hypertension and hypercholesterolaemia practice guidelines. Also, it is known that workload in public primary healthcare clinics in Malaysia is so high that little time is left for meaningful interactions between patients and providers (Risso-Gill *et al*., 2015). Therefore, encouraging proactive involvement of other health professionals such as allied health allows more time for providers to strengthen patient–provider relationships and improve knowledge transfer to patients. With these multifaceted approaches, it is hypothesised that the EnPHC intervention will enhance adherence to clinic appointments and treatment and improve self-management practices and patient-reported experience at the patient level. This will subsequently improve disease control and can be measured through intermediate clinical outcomes such as HbA1c, blood pressure and lipid levels.

Hypercholesterolaemia was among the NCDs that were shown to be under-diagnosed and suboptimally managed in Phase 1 of the Malaysian Health Systems Research (MHSR) report (Ministry of Health Malaysia and Harvard University TH Chan School of Public Health, 2016). EnPHC-EVA: Facility study was designed to include a sub-sample of hypercholesterolaemia patients but this plan had to be dropped because there was no means to identify a list of patients diagnosed with hypercholesterolaemia. Patient visits for T2DM and hypertension, on the other hand, had defined appointment registers for each clinic work day. However, T2DM and hypertension patients with hypercholesterolaemia as comorbidity were captured.

Process evaluation of EnPHC interventions in the primary healthcare clinic is conducted by a separate study team to improve execution of the interventions. This, together with an impact evaluation done at both the community and facility level, will complement each other and allow for triangulation of findings. Only then, the intervention can be refined and replicated for further nationwide scale up.

The strengths of this study include the presence of a control group, random intervention allocation and repeated data points which maintained internal validity. Furthermore, the clusters were matched to minimise bias that can exist from differences in clinic composition. An intervention checklist was also incorporated to adjust for the degree of intervention implementation within each clinic and explain potential contamination or incomplete implementation in the control and intervention clinics, respectively. In addition, the transparency in reporting this study by adhering to the Transparent Reporting of Evaluations with Nonrandomized Designs (TREND) statement is a form of knowledge sharing and contributes to rigorous evidence to guide future research and practice (Des Jarlais *et al*., 2004). Another strength of this study is that its implementation and evaluation teams were from separate institutions, the former is a planning and governing division for public health clinics while the latter is a research organisation. This maintained objectivity of the study and minimises the risk of publication and outcome-reporting bias. Lastly, this study also captures potential unintended effects on the healthcare staff, measured as change in health provider job satisfaction following the implementation of EnPHC through the provider survey.

There are several limitations in this study. First, it is not possible to distinguish the effects from each activity of the intervention as it is a complex intervention. Next, the findings of this study will only be generalisable to clinics with similar characteristics. Long-term outcomes such as micro- and macrovascular complications and behavioural changes could not be measured as the study duration is not sufficient to observe these changes. Missing data arising from untraceable records and incomplete documentation in medical records may introduce bias to the findings. This could be improved by integrated electronic information systems in the long run.

In summary, this study will provide evidence on the effectiveness of a complex intervention designed to reduce the number of undiagnosed T2DM and hypertension patients as well as improve the overall quality of care in this group of patients to delay overall disease progression.

## Supplementary material

For supplementary material accompanying this paper visit http://dx.doi.org/10.1017/S1463423620000250. click here to view supplementary material
