# Supplementary material for: Study protocol on Enhanced Primary Healthcare (EnPHC) interventions: a quasi-experimental controlled study on diabetes and hypertension management in primary healthcare clinics
Source: Prim Health Care Res Dev. 2020 Aug 13;21:e27. doi: 10.1017/S1463423620000250 (PMC7443798; doi:10.1017/S1463423620000250)
Supplement: Supplementary file 1 [file S1463423620000250sup001.docx]

**APPENDIX A – Data Extraction Form for Retrospective Chart Review**


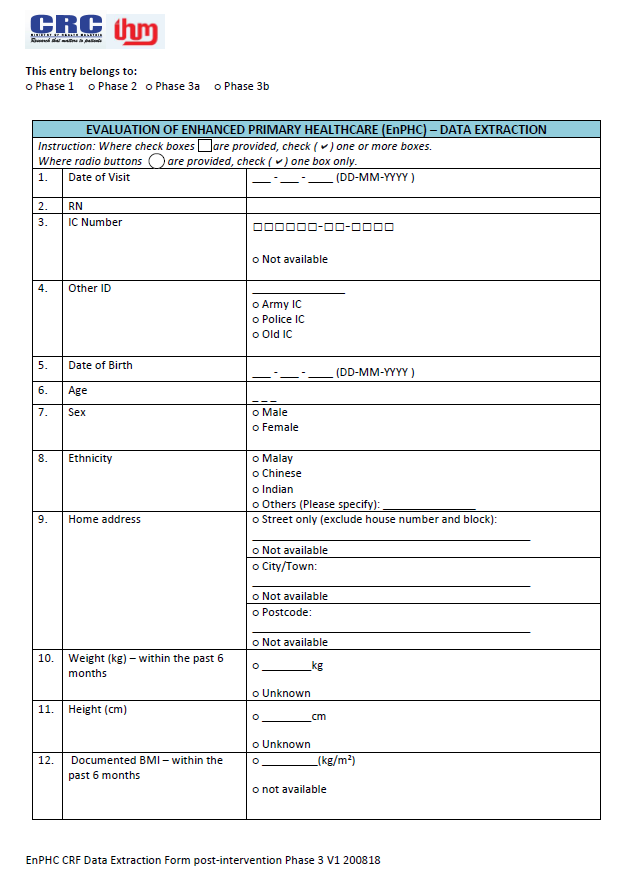

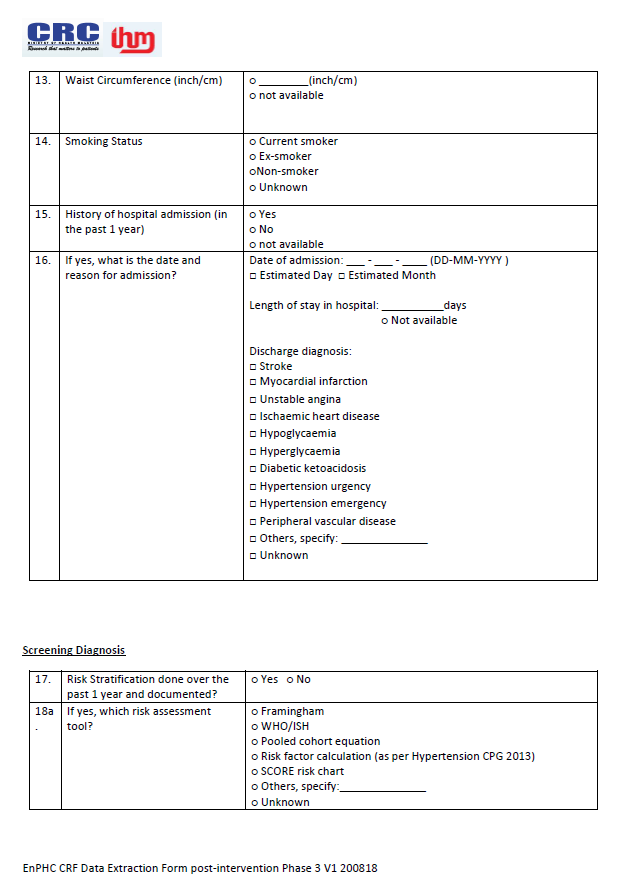


**Appendix A (Continued)**


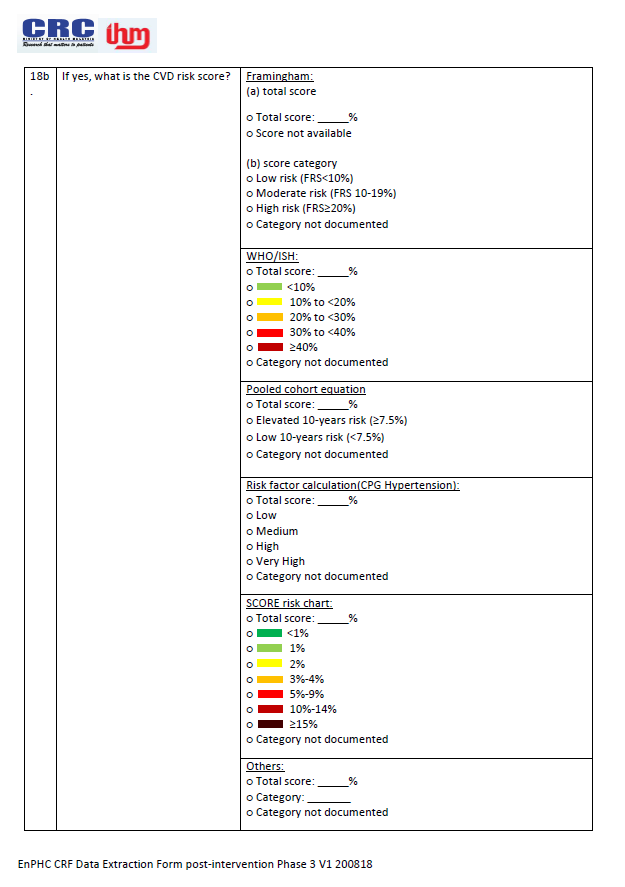

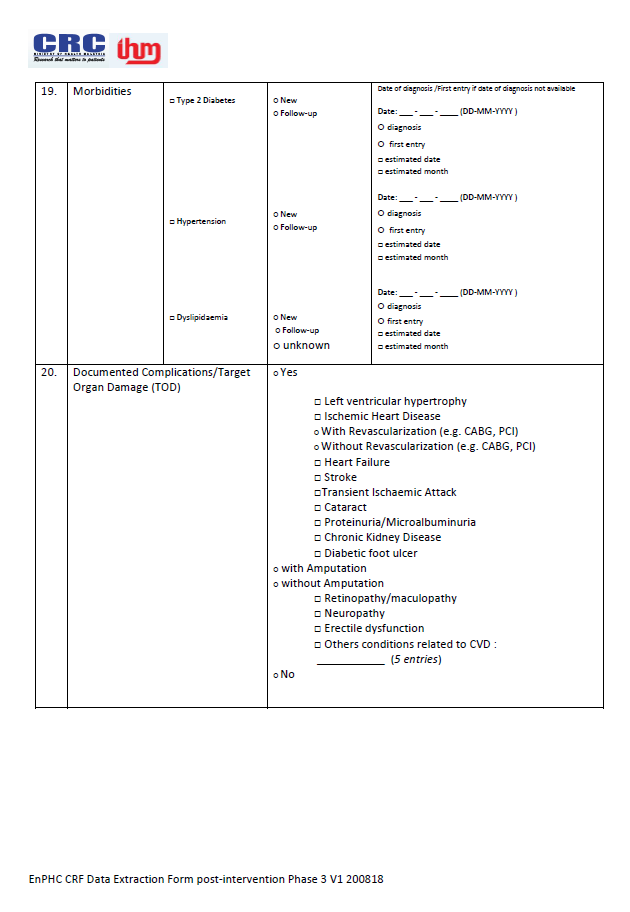


**Appendix A (Continued)**


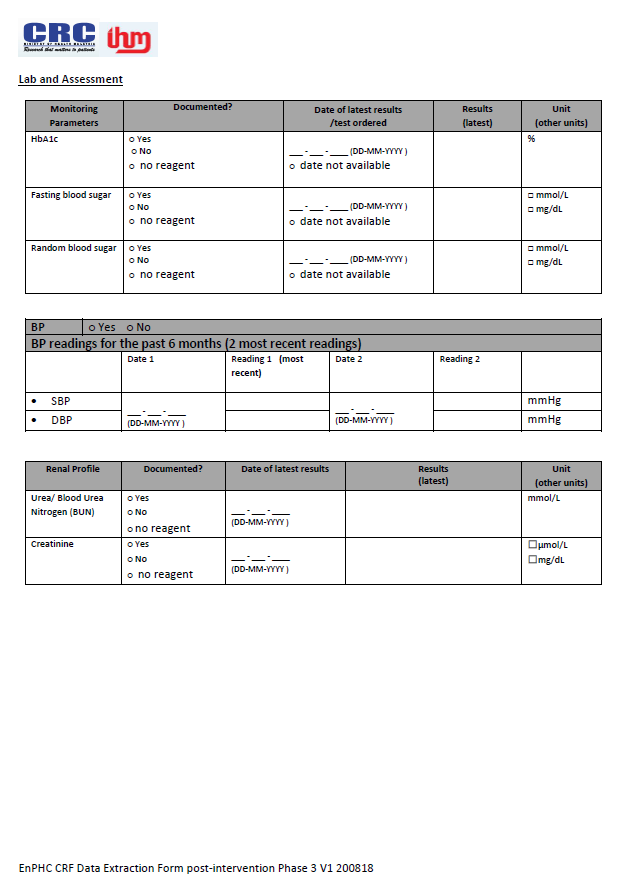

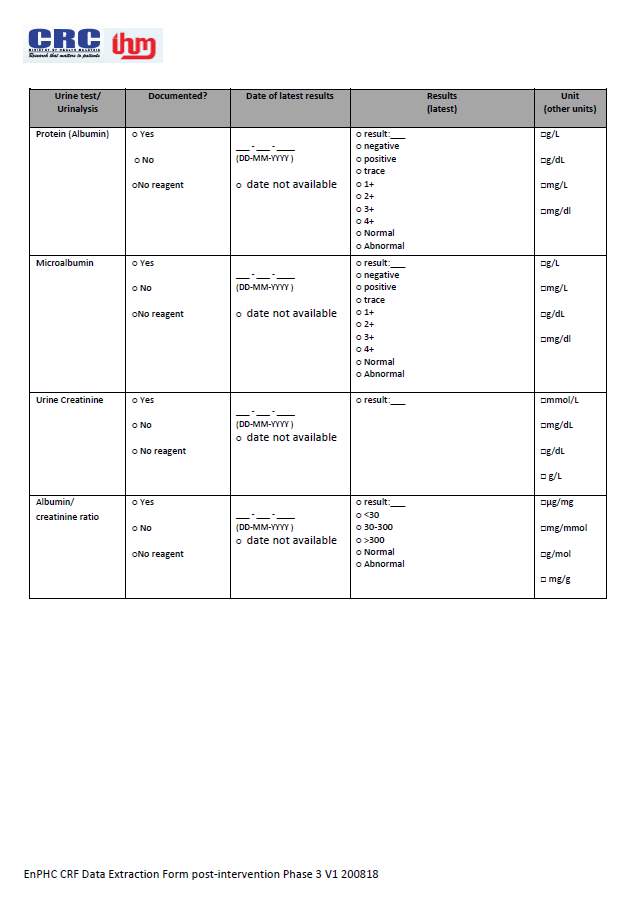


**Appendix A (Continued)**


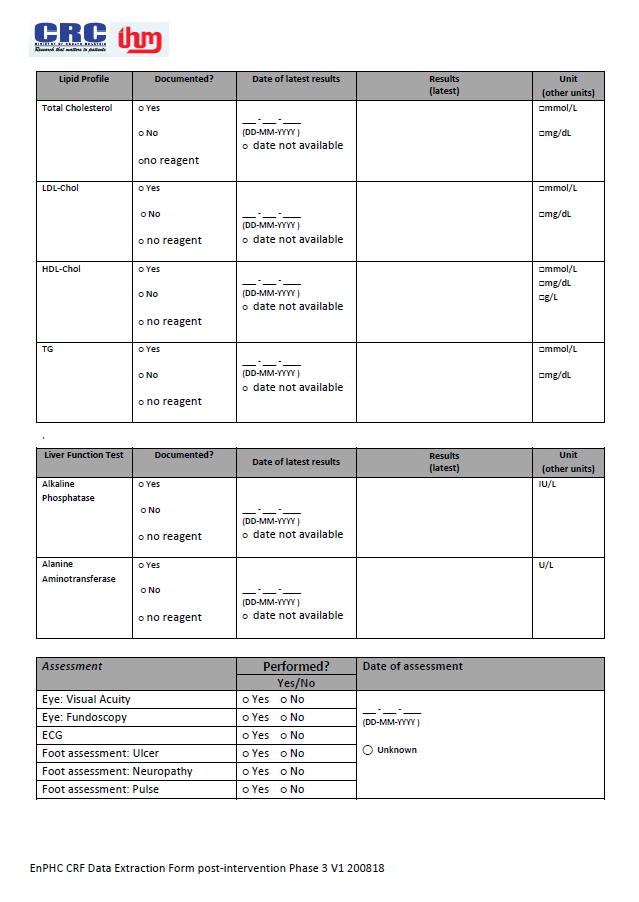

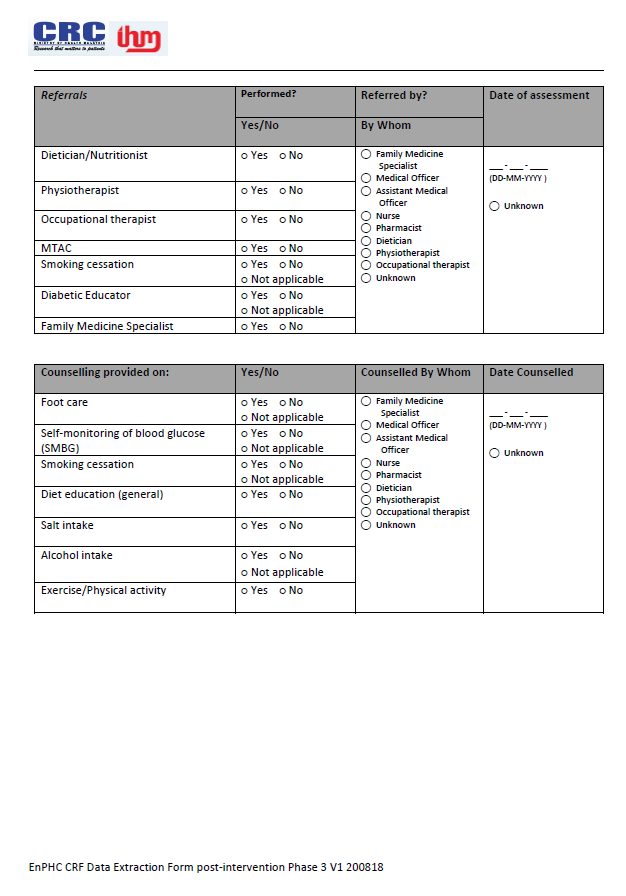


**Appendix A (Continued)**


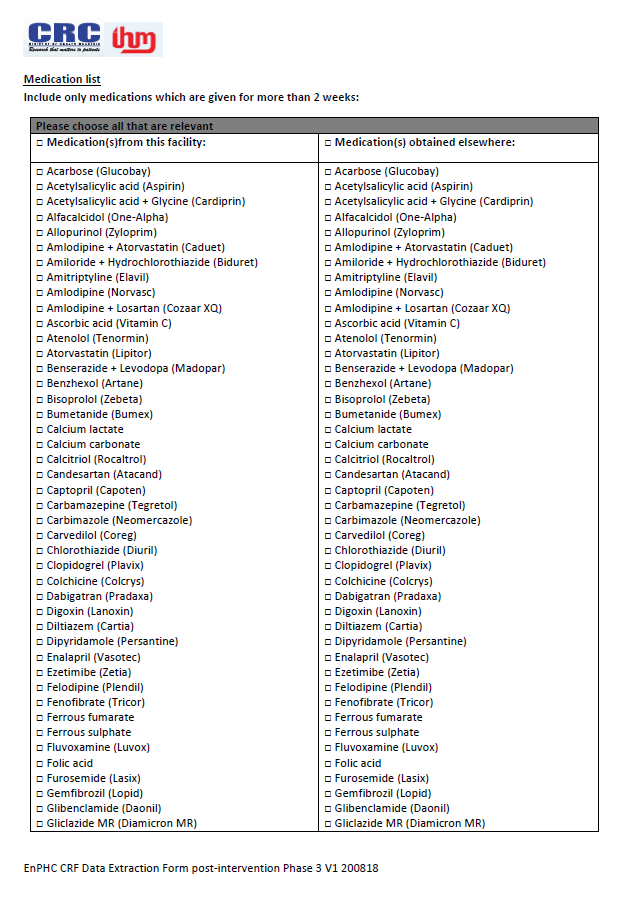

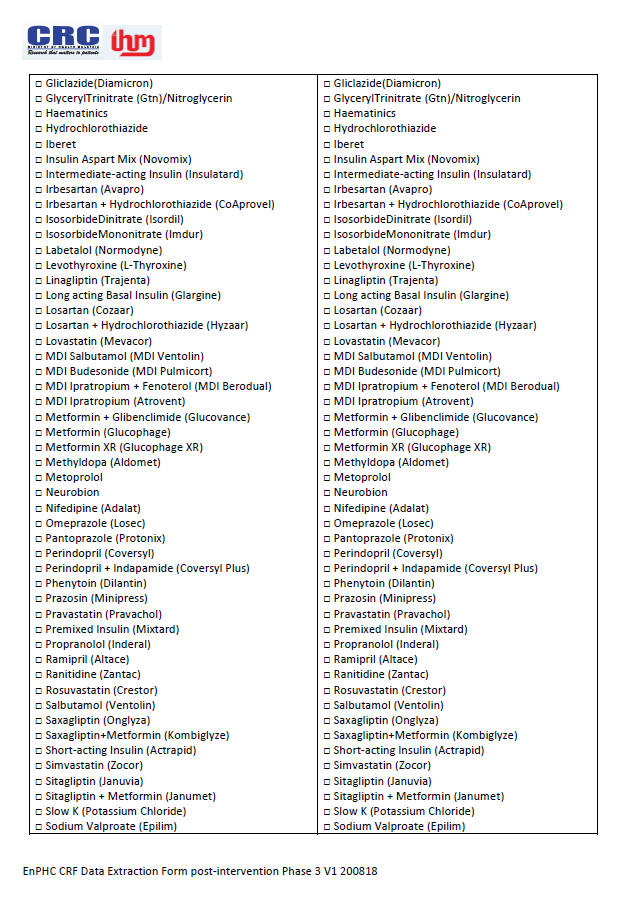


**Appendix A (Continued)**


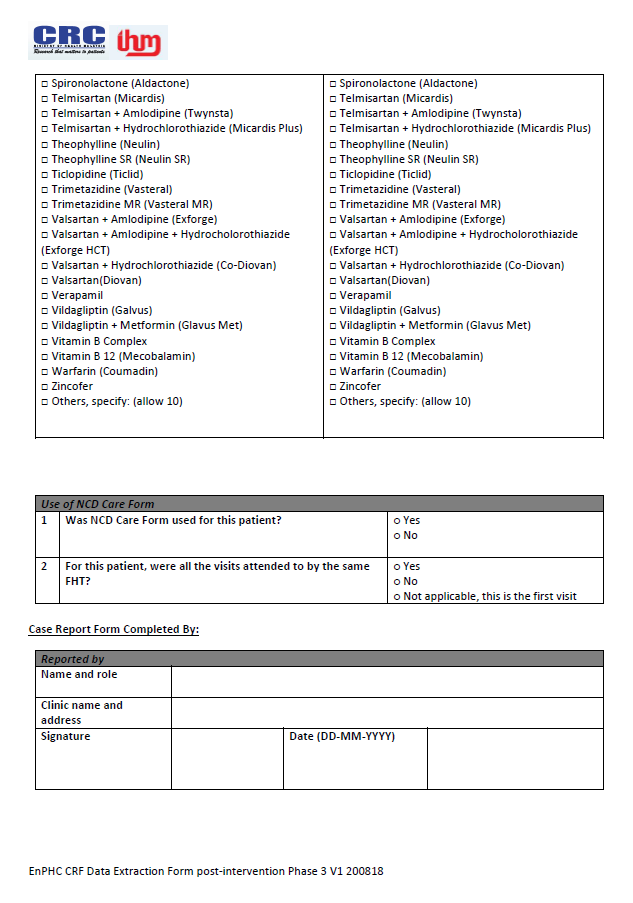


**Appendix B – Patient Exit Questionnaire**


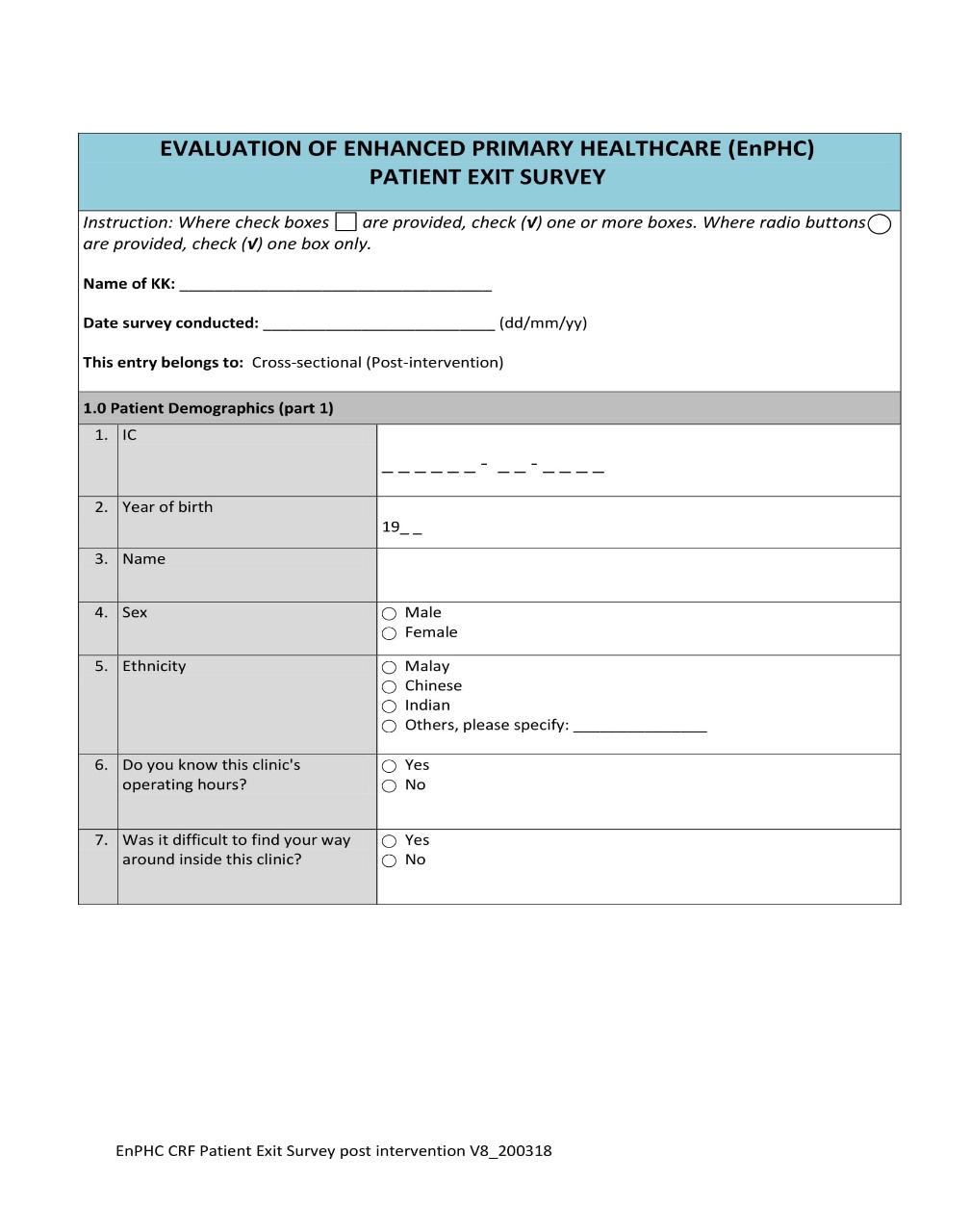

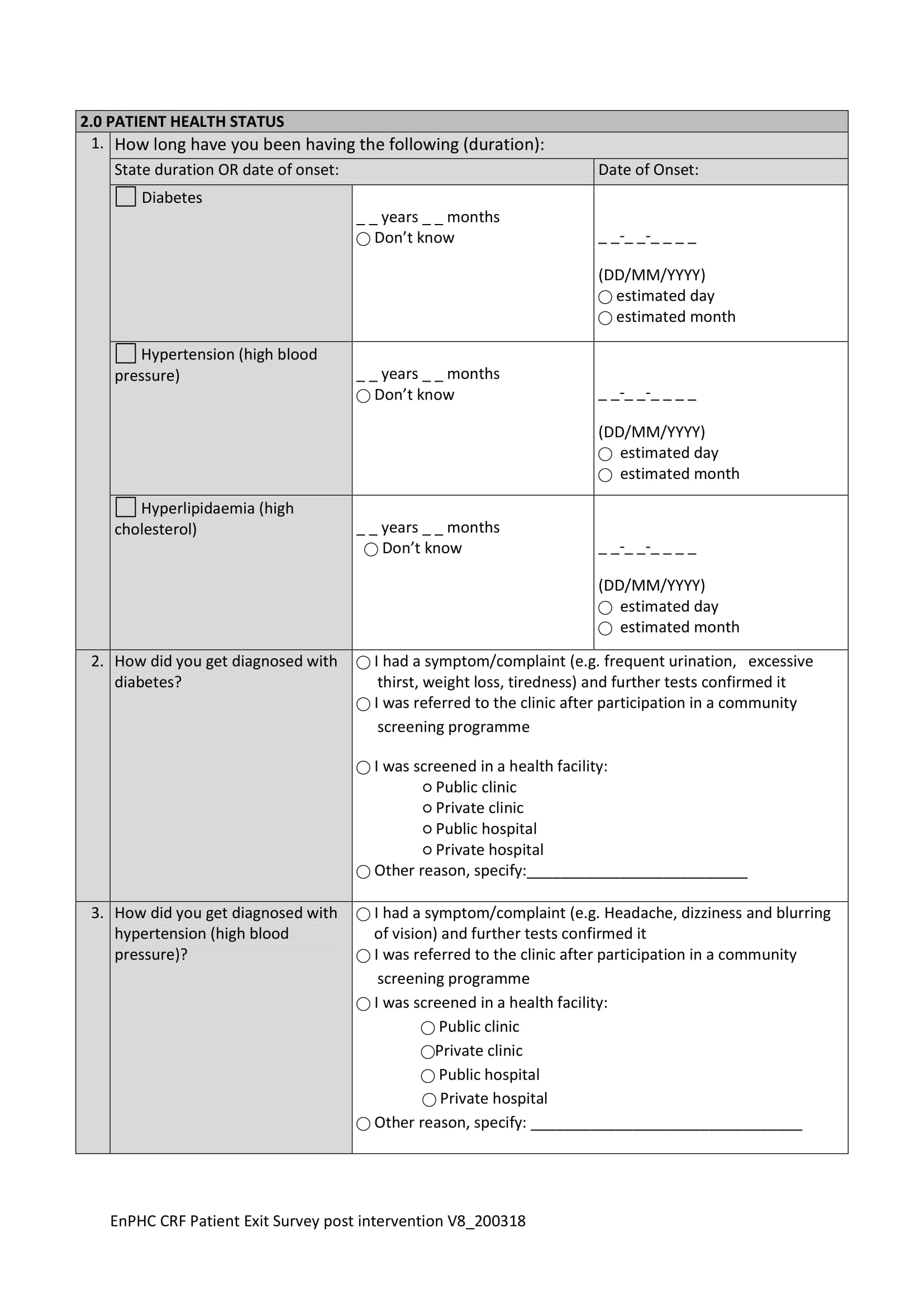


**Appendix B – Patient Exit Questionnaire (Continued)**


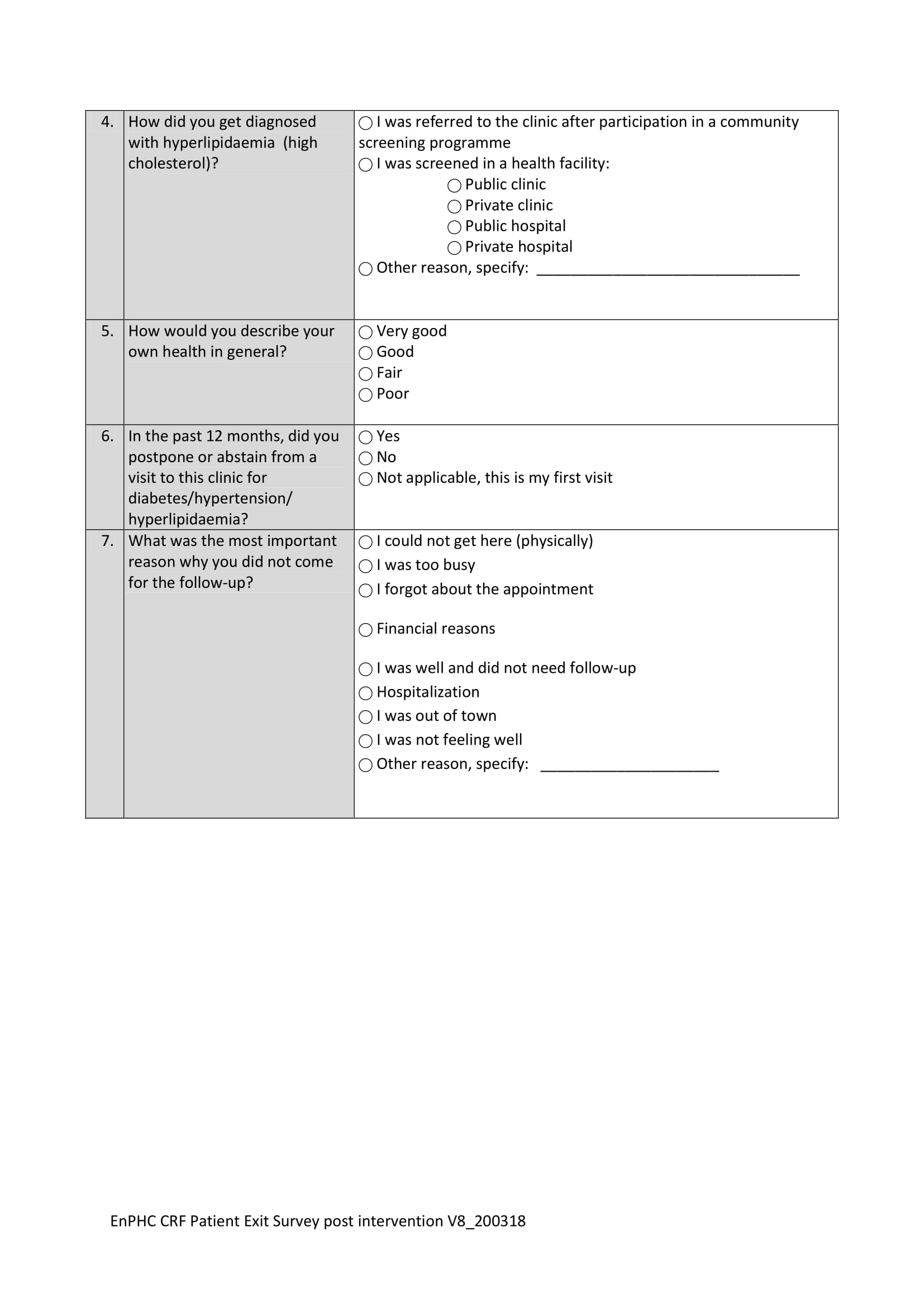

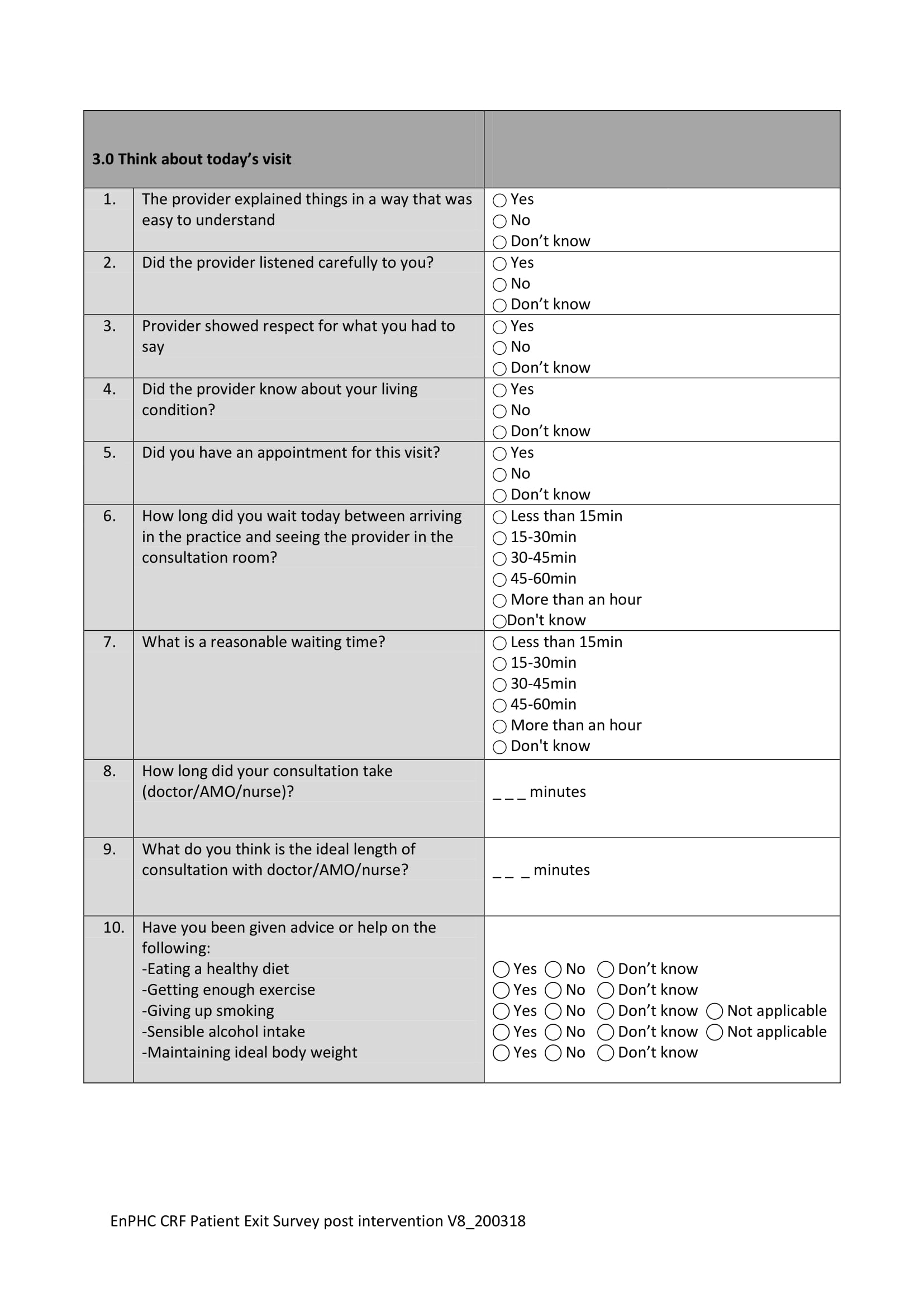


**Appendix B – Patient Exit Questionnaire (Continued)**


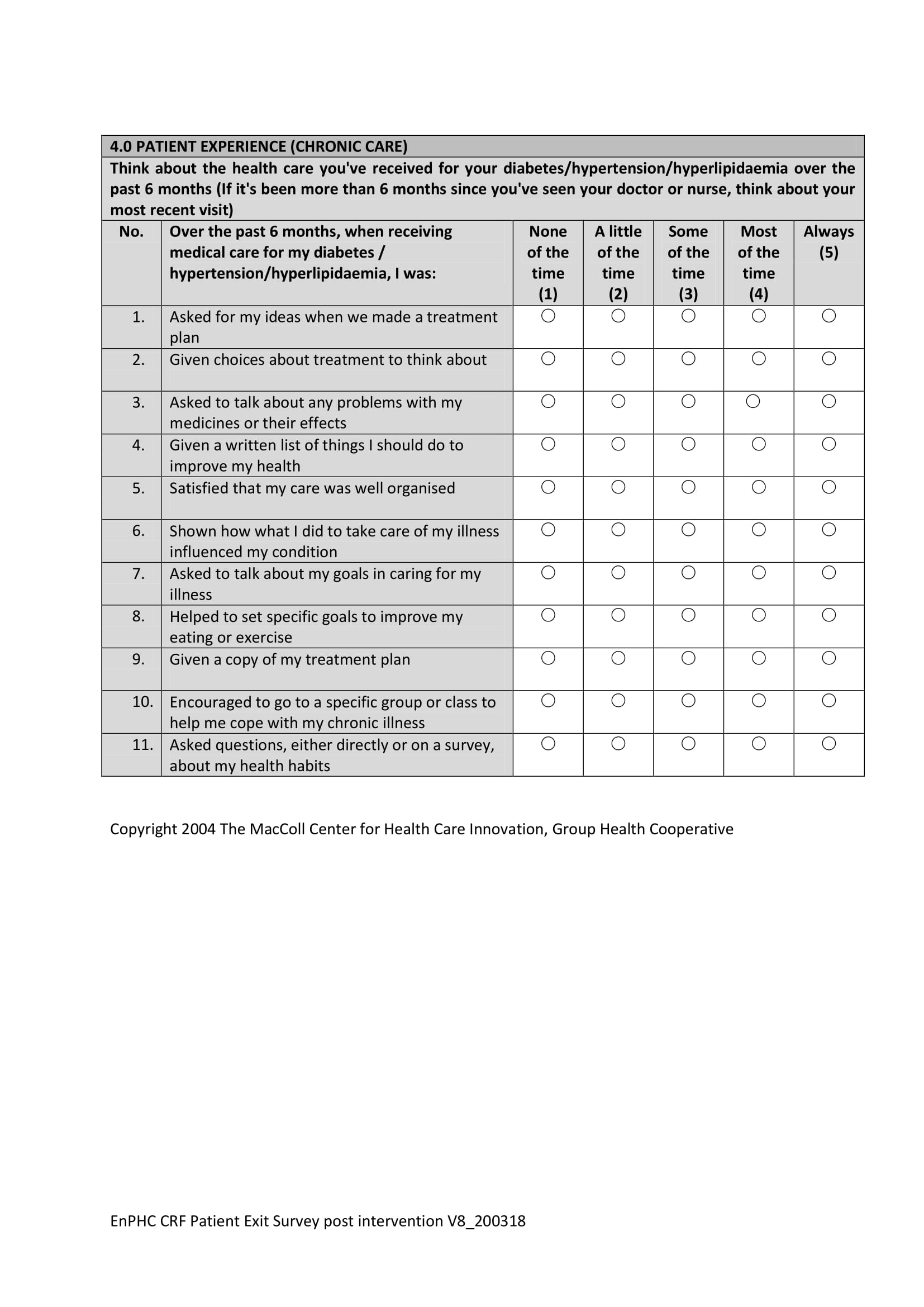

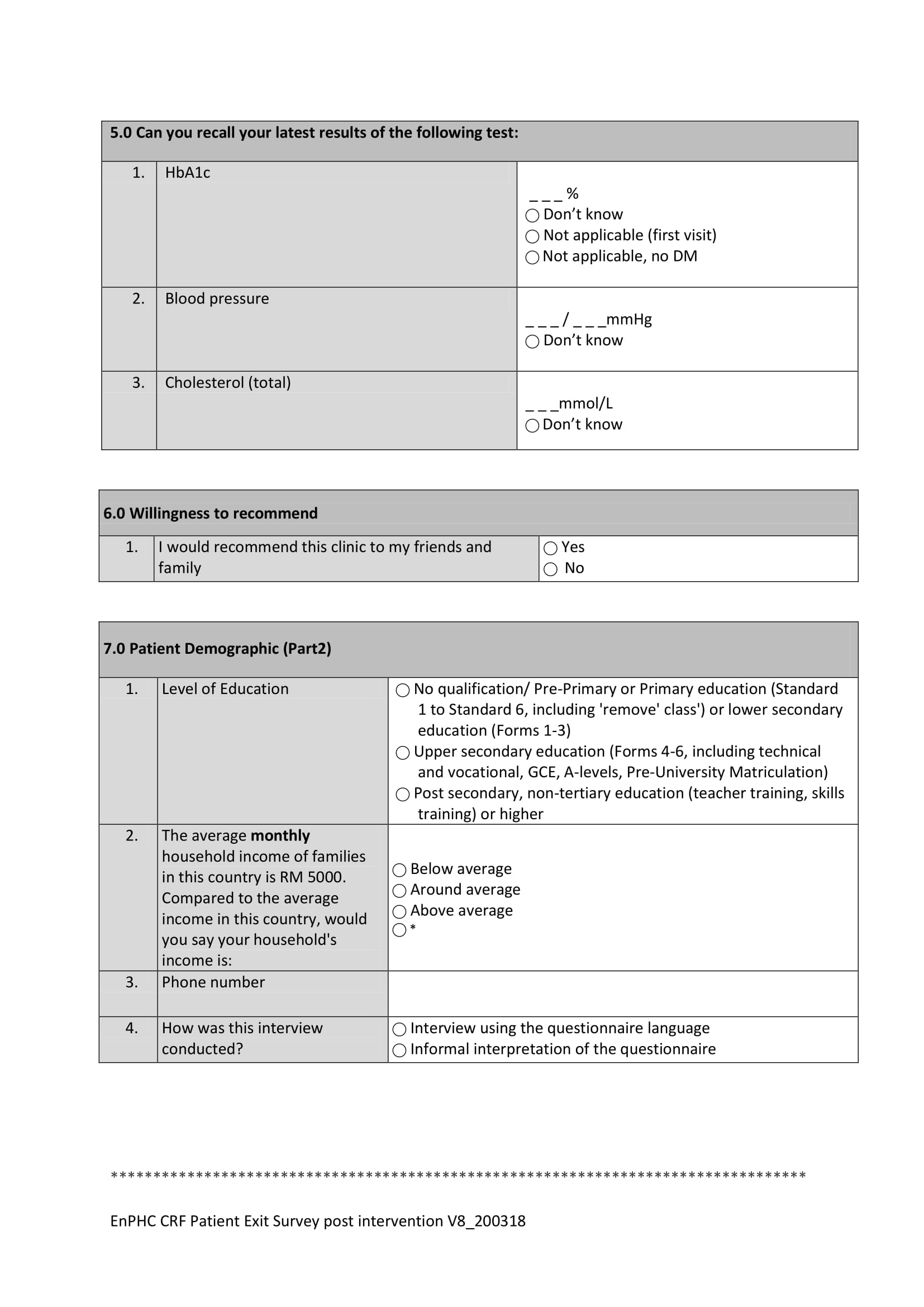


**Appendix B – Patient Exit Questionnaire (Continued)**


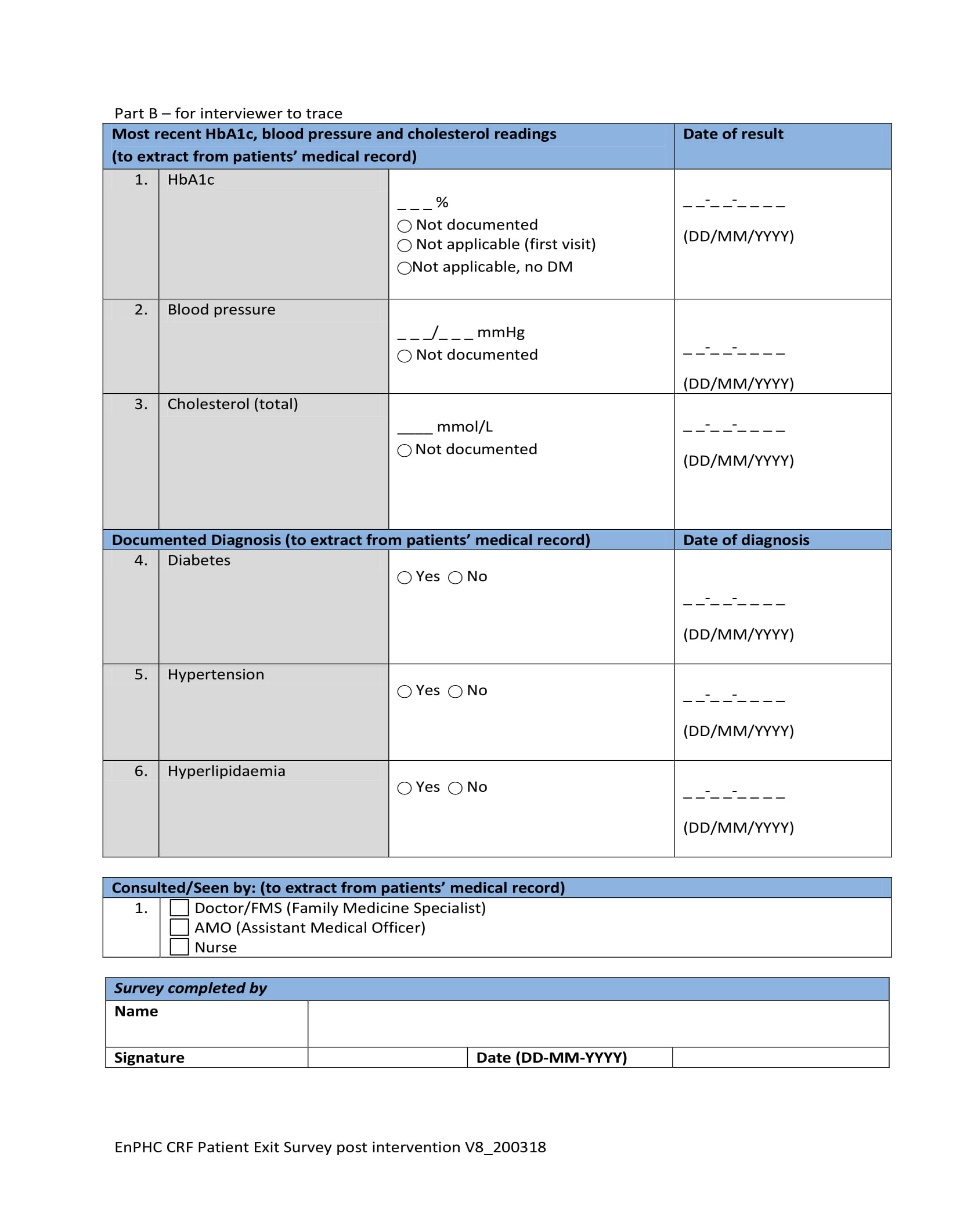


**Appendix C – Provider Questionnaire**


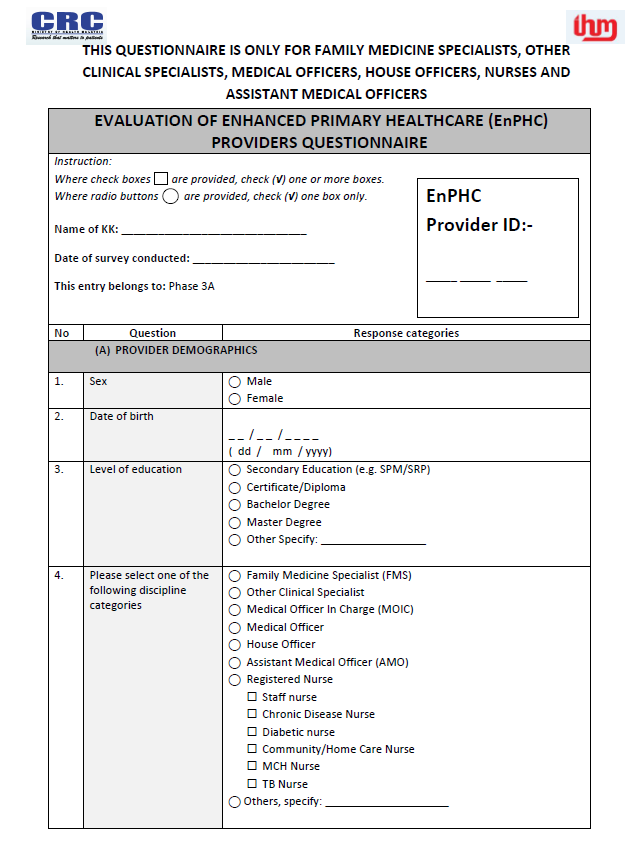

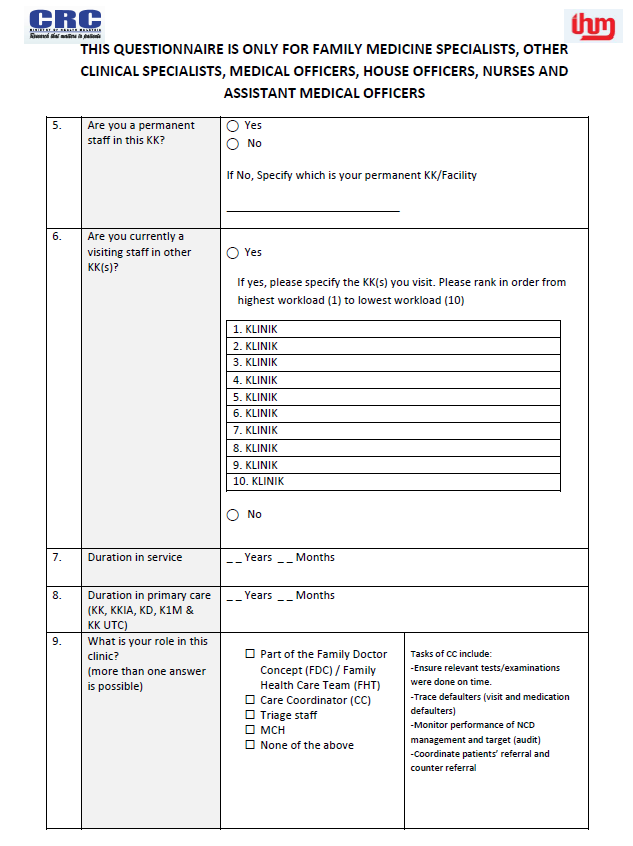


**Appendix C – Provider Questionnaire (Continued)**


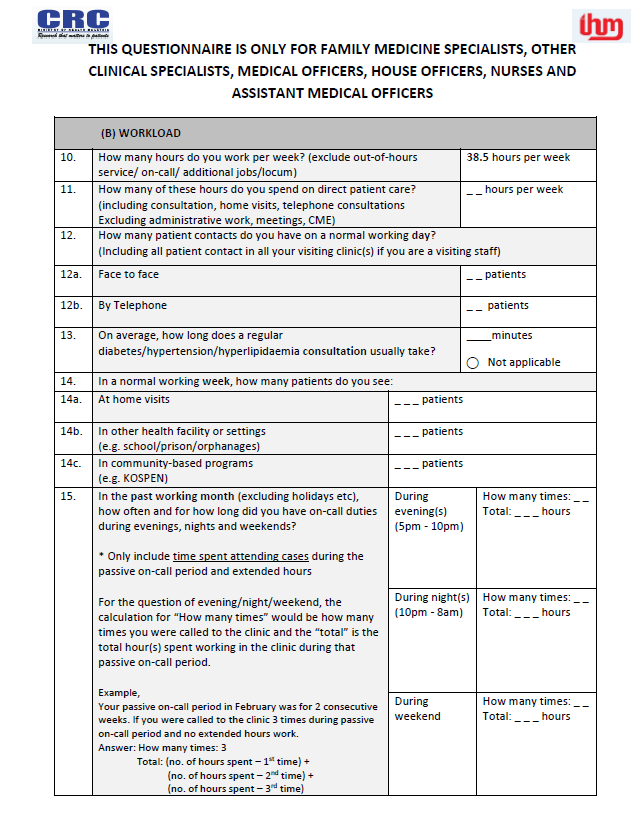

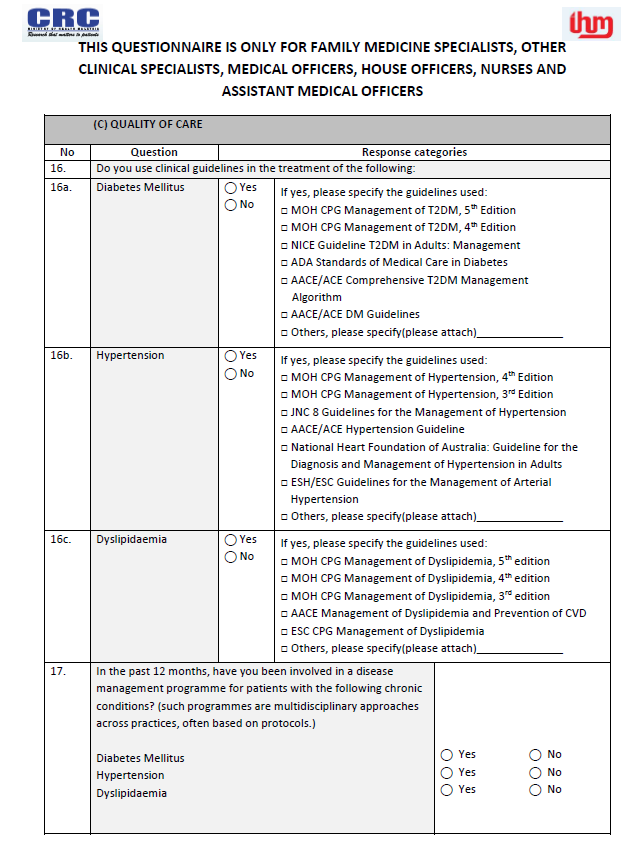


**Appendix C – Provider Questionnaire (Continued)**


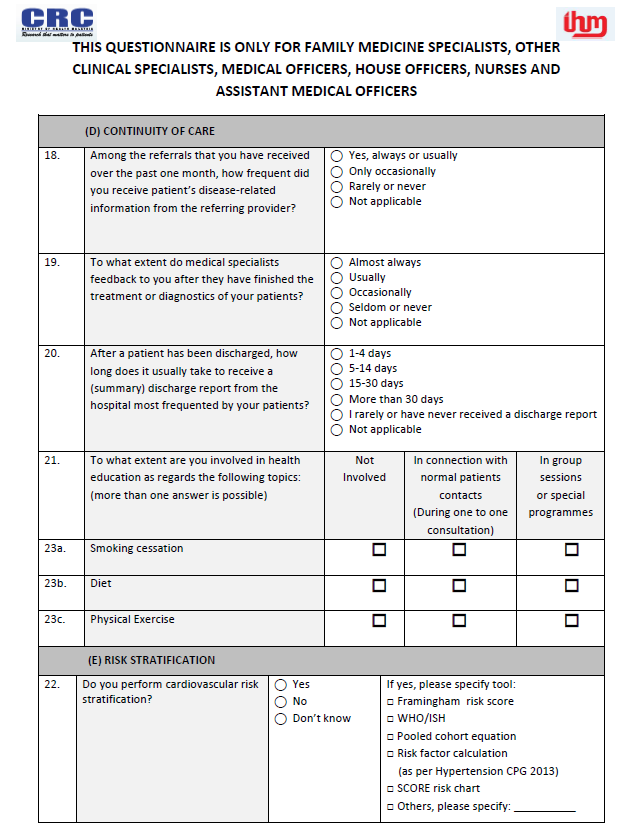

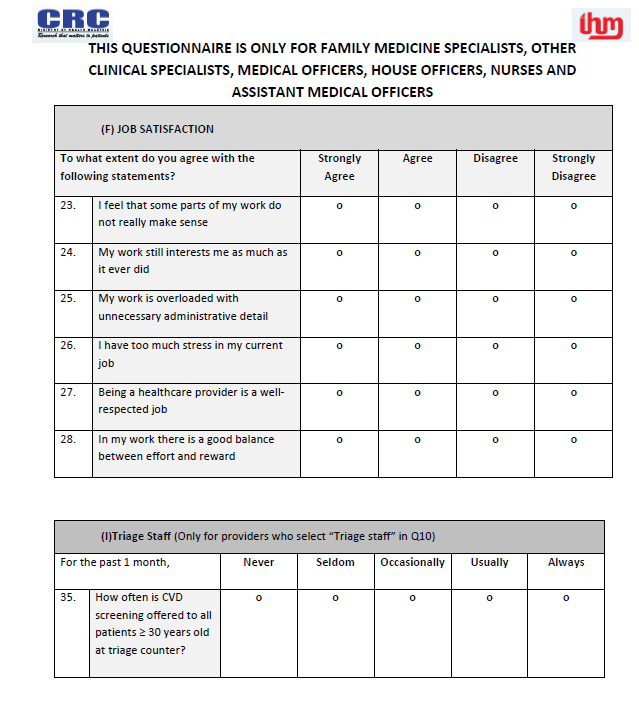


**Appendix D – Facility Questionnaire**


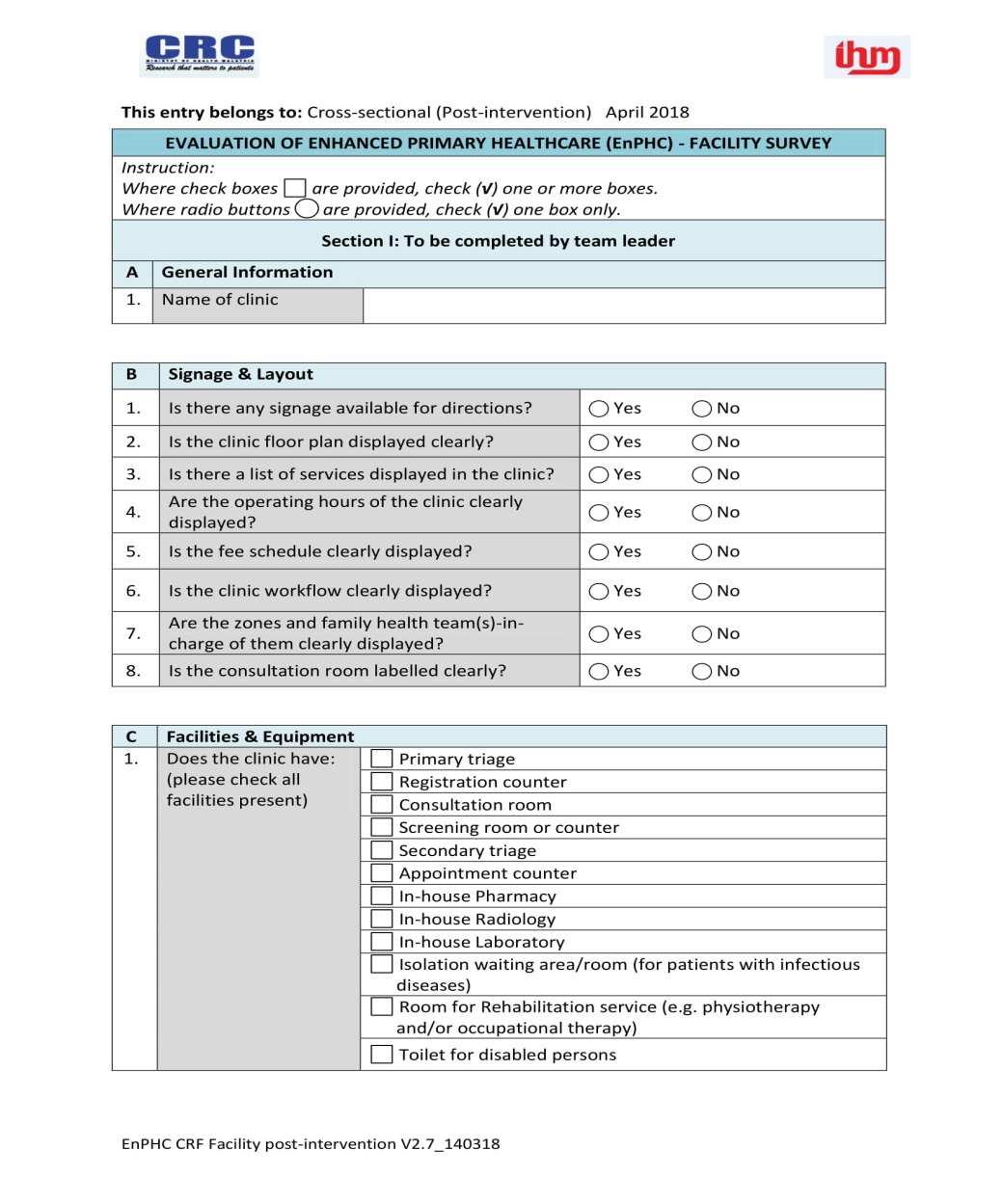
**
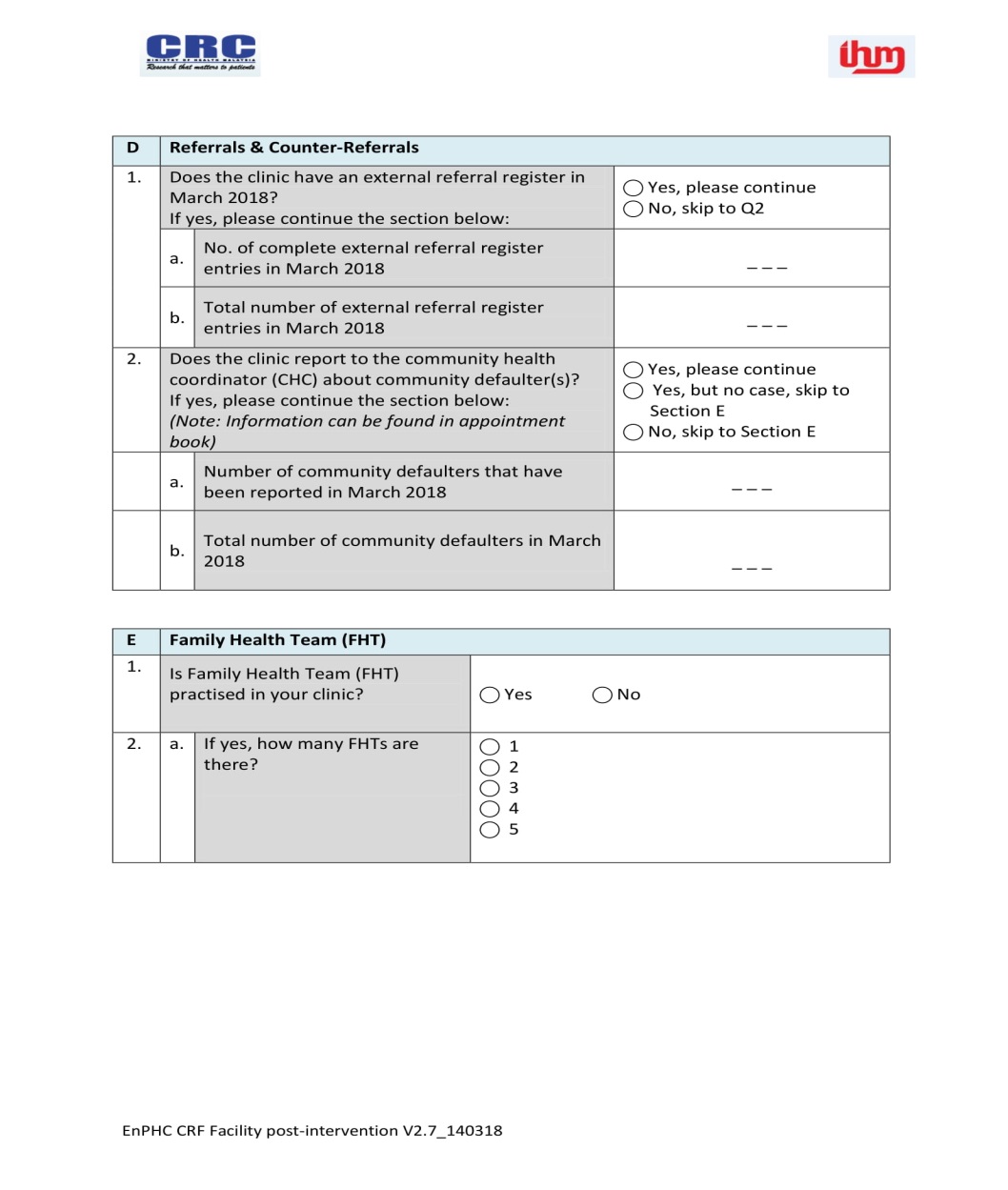
**

**Appendix D – Facility Questionnaire (Continued)**


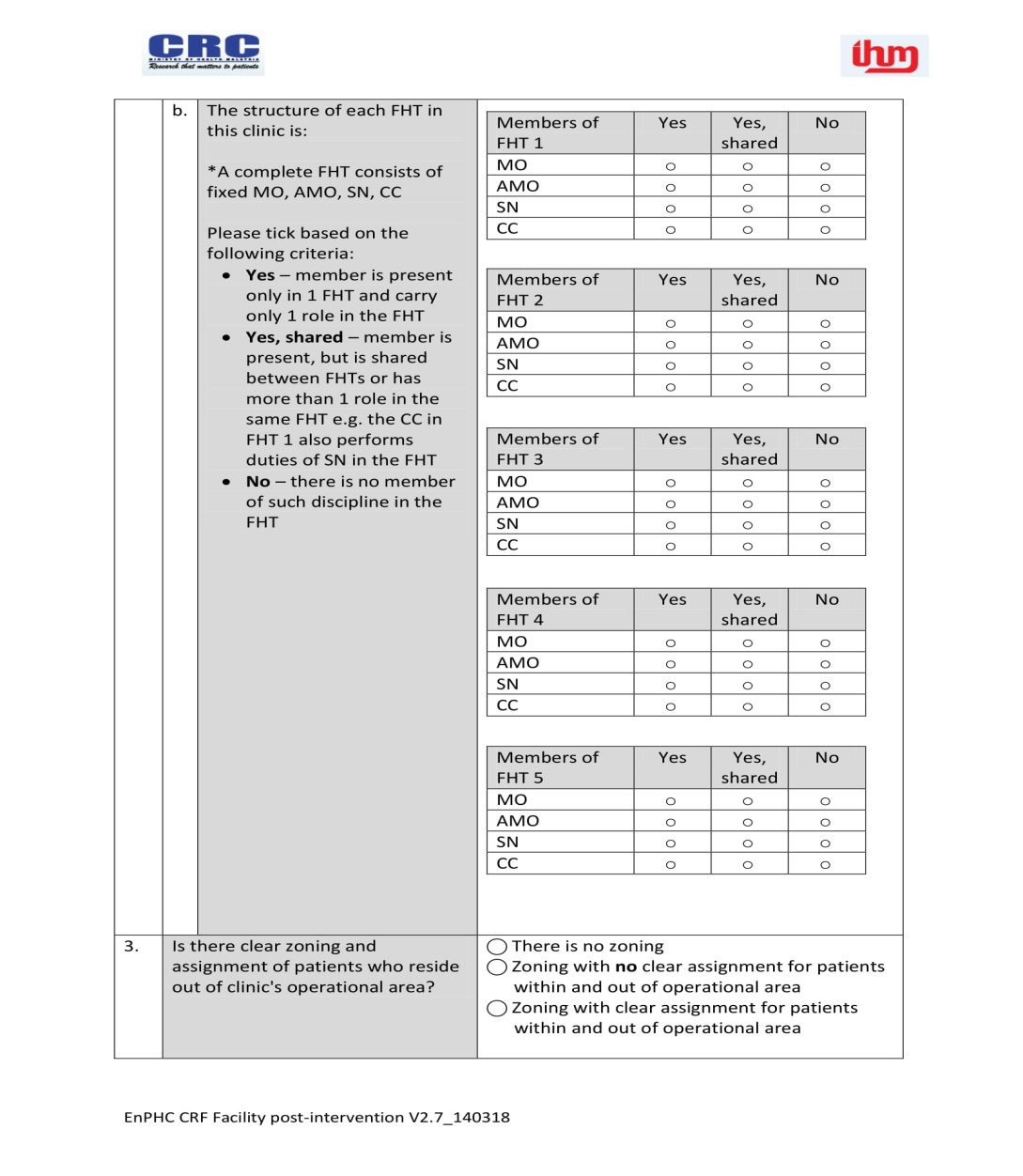

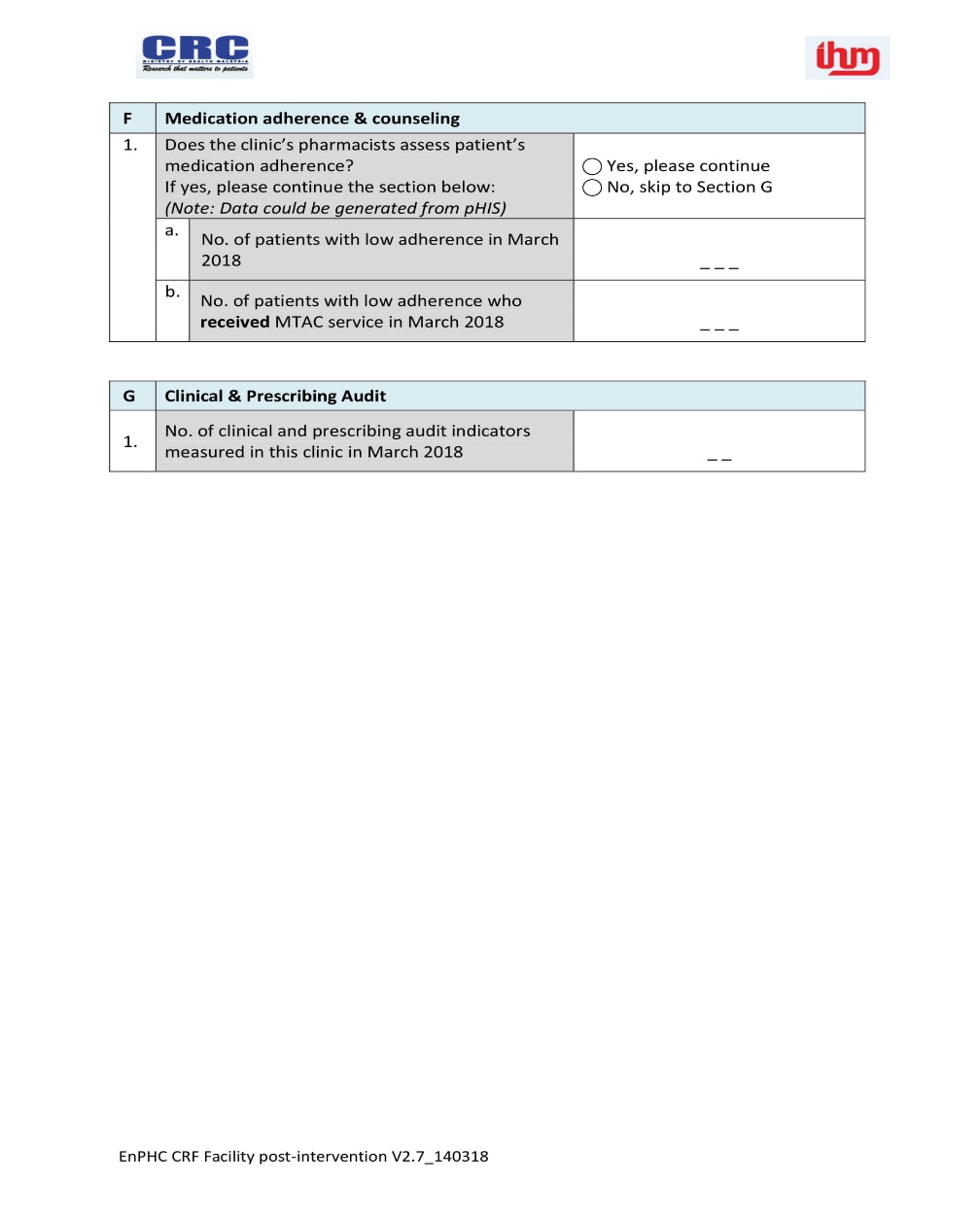


**Appendix D – Facility Questionnaire (Continued)**


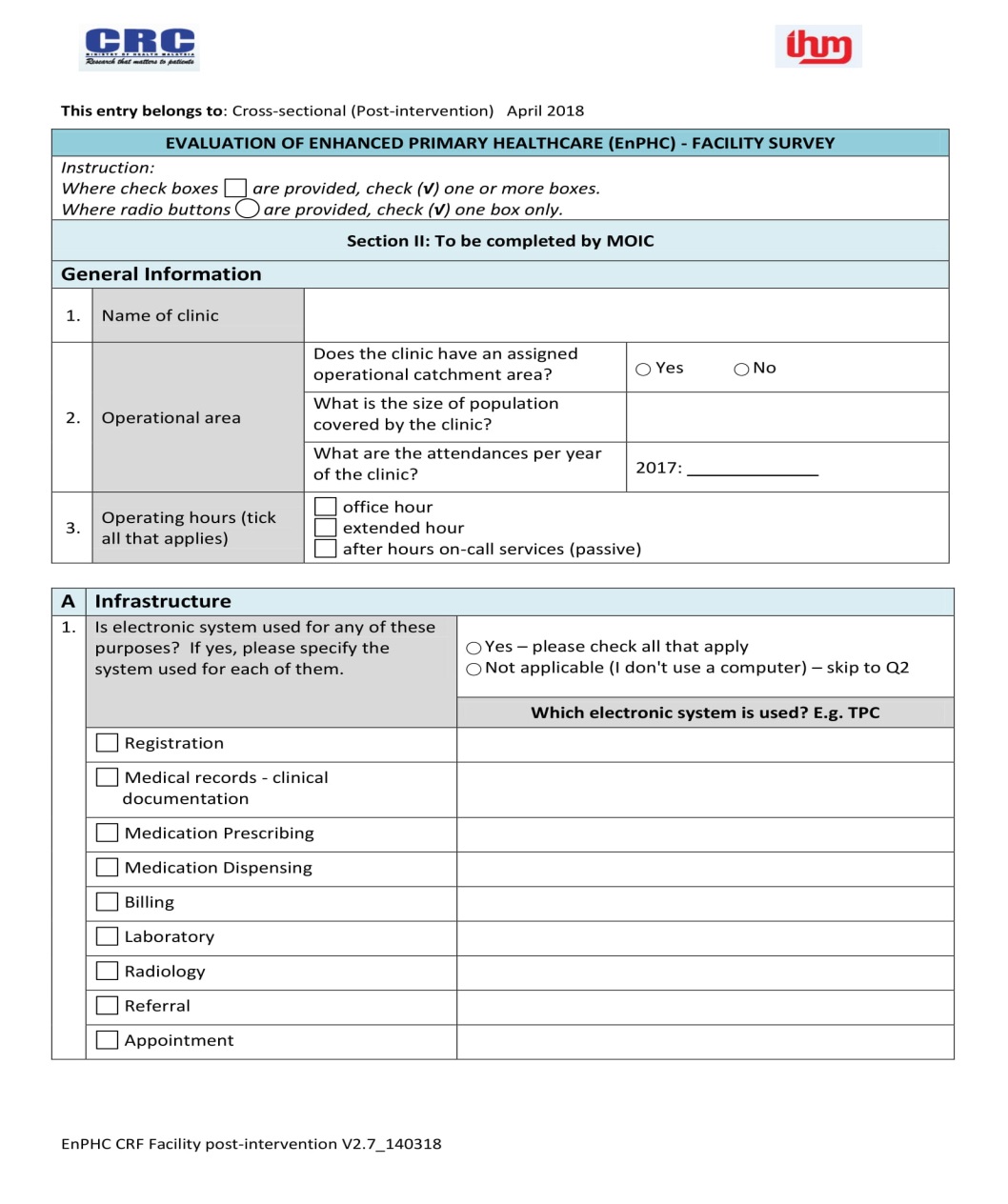

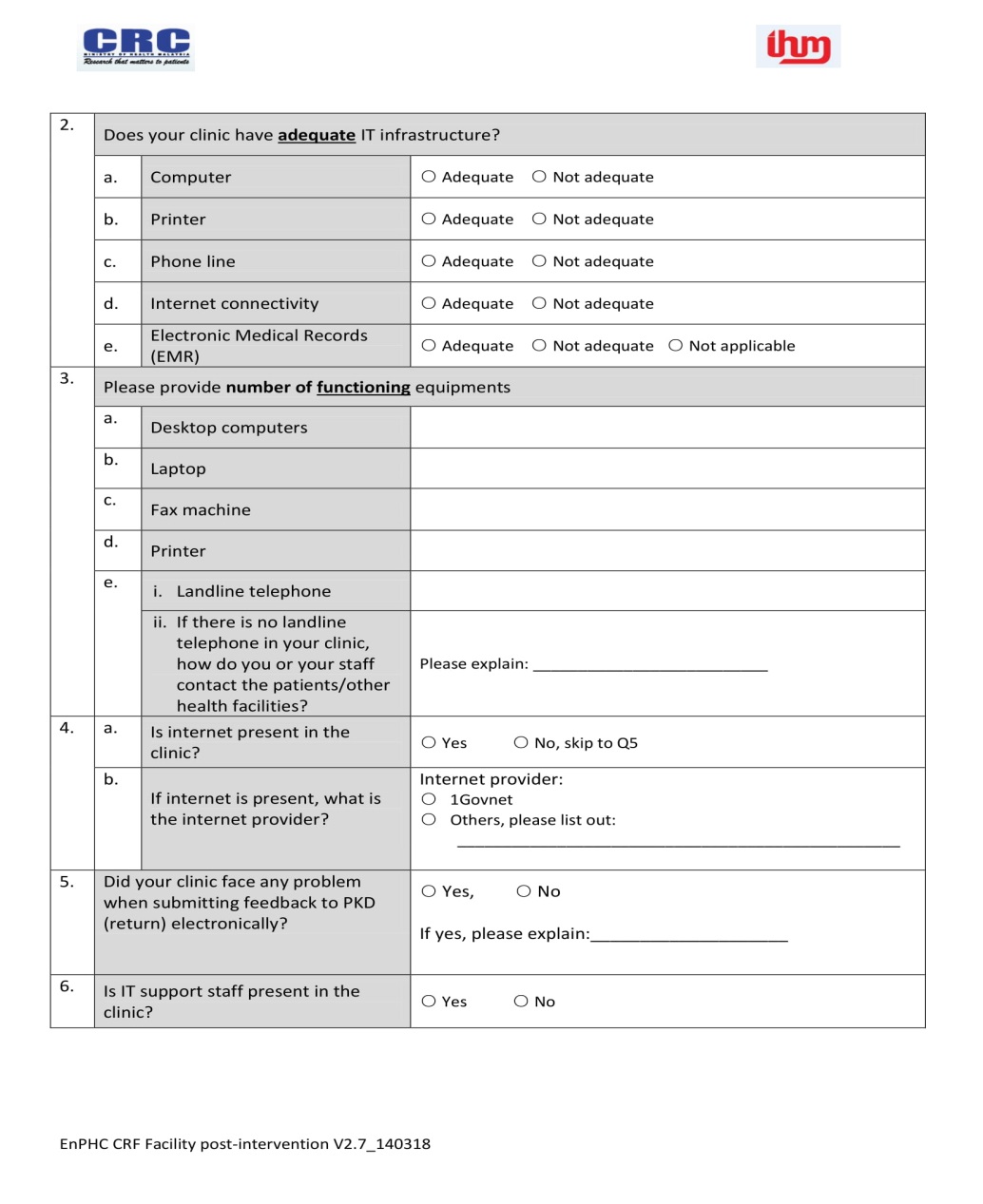


**Appendix D – Facility Questionnaire (Continued)**


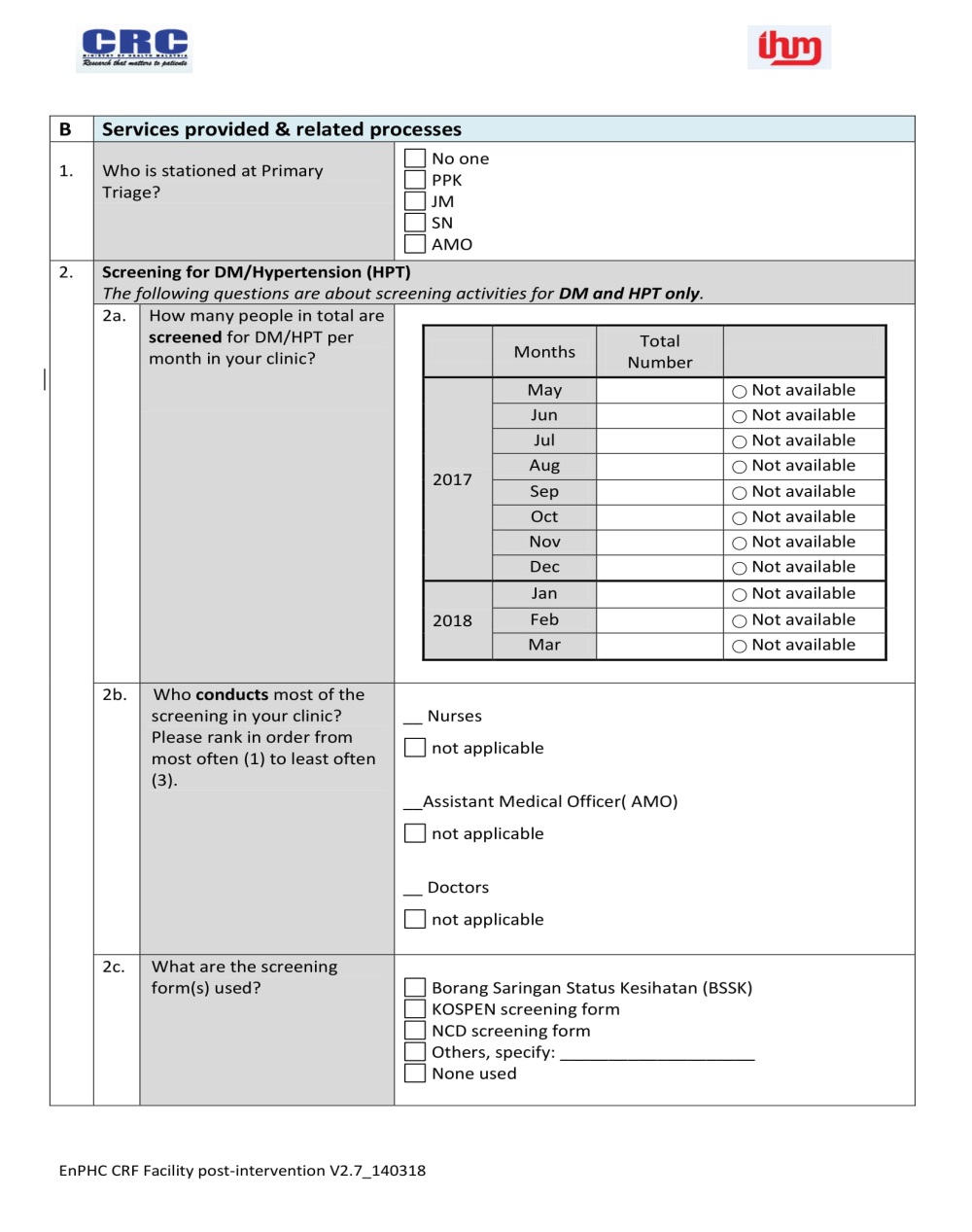

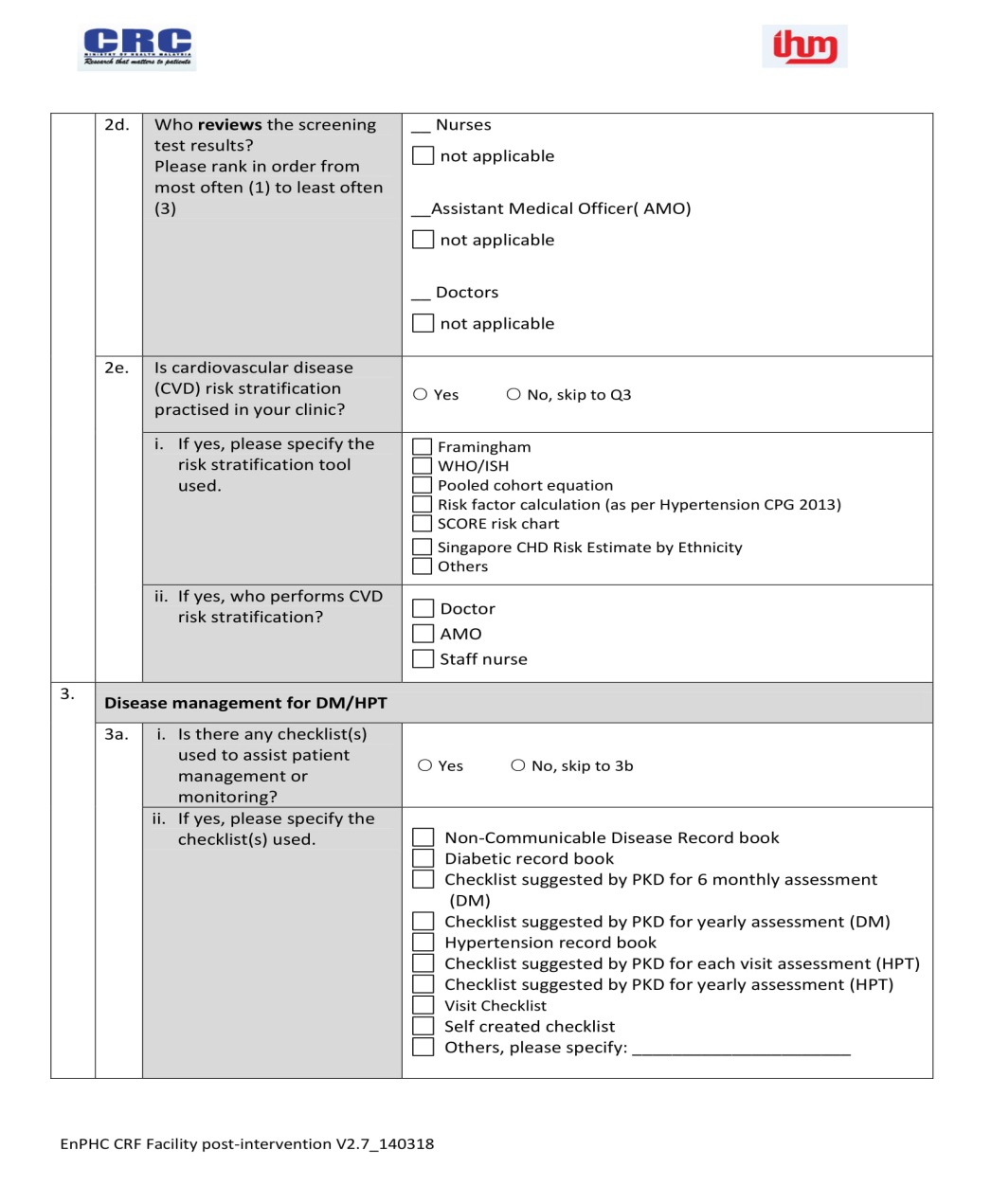


**Appendix D – Facility Questionnaire (Continued)**


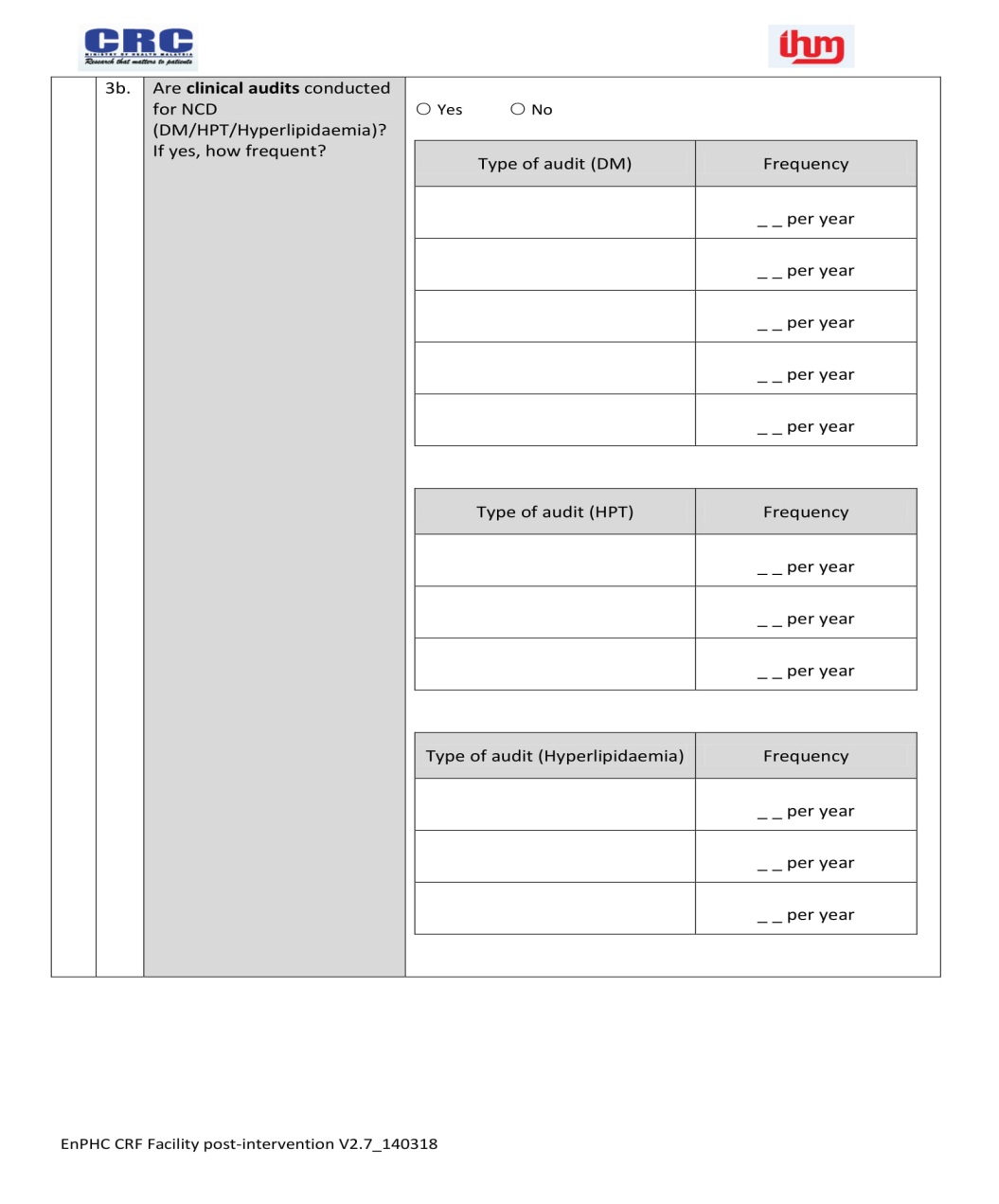

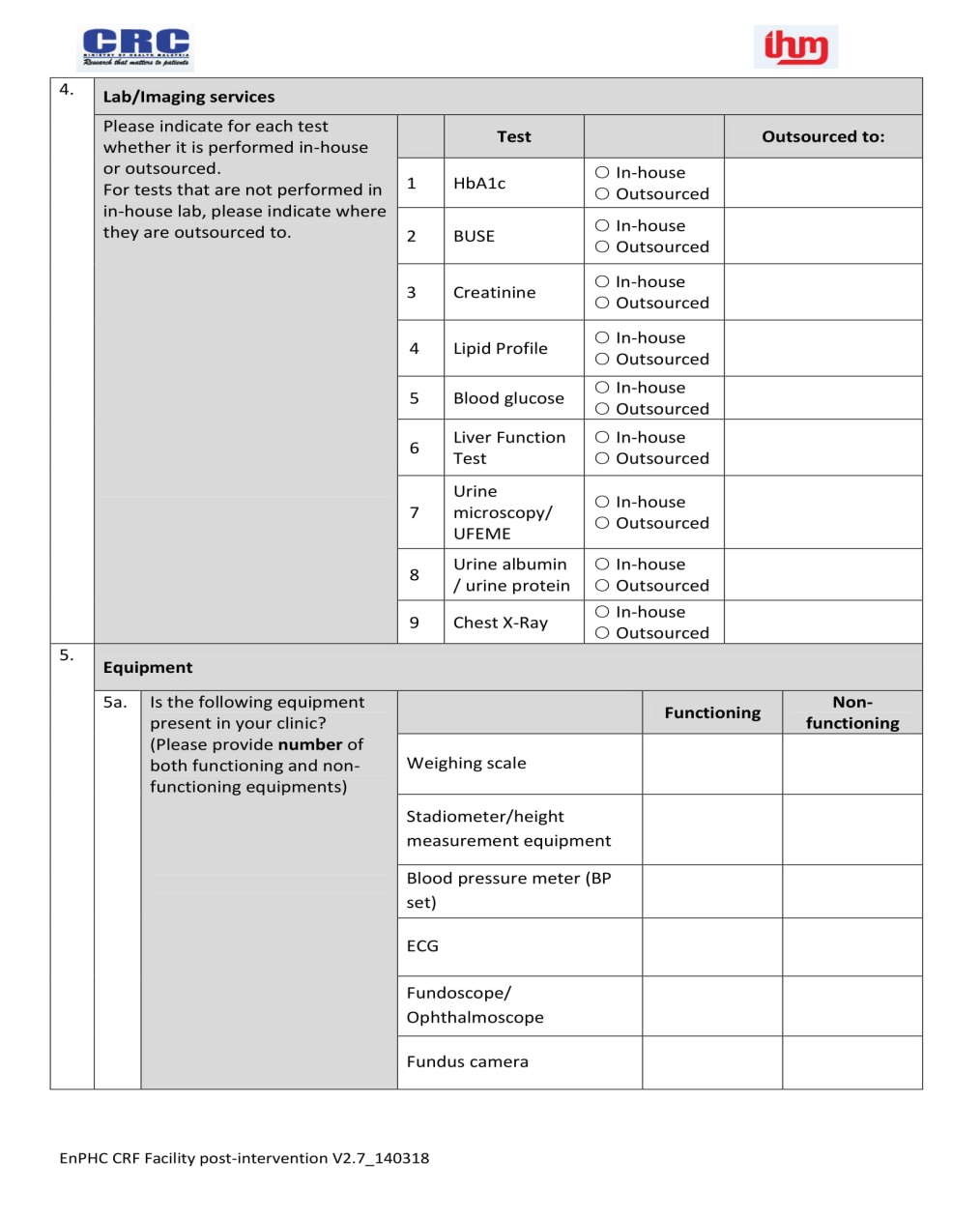


**Appendix D – Facility Questionnaire (Continued)**


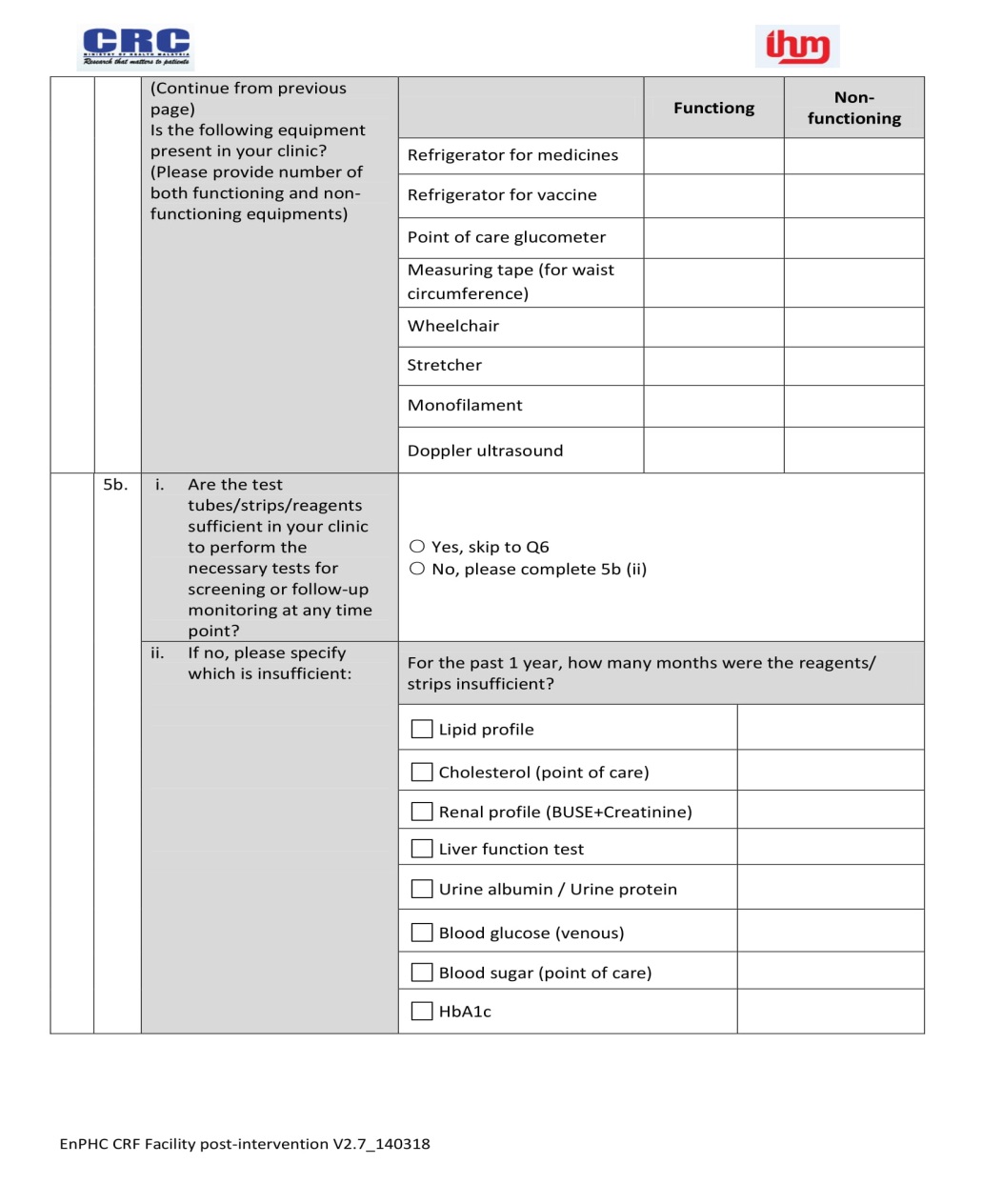

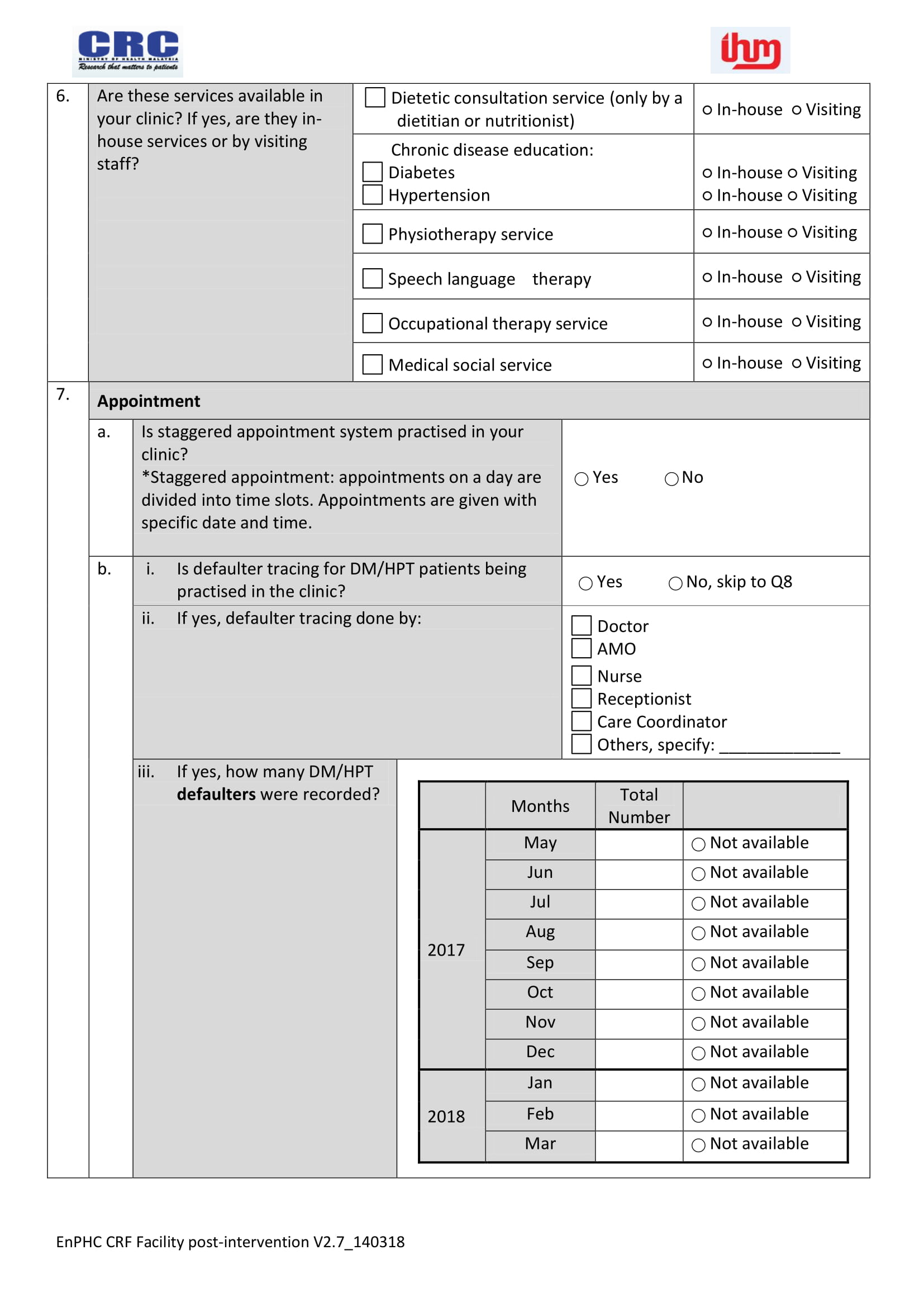


**Appendix D – Facility Questionnaire (Continued)**


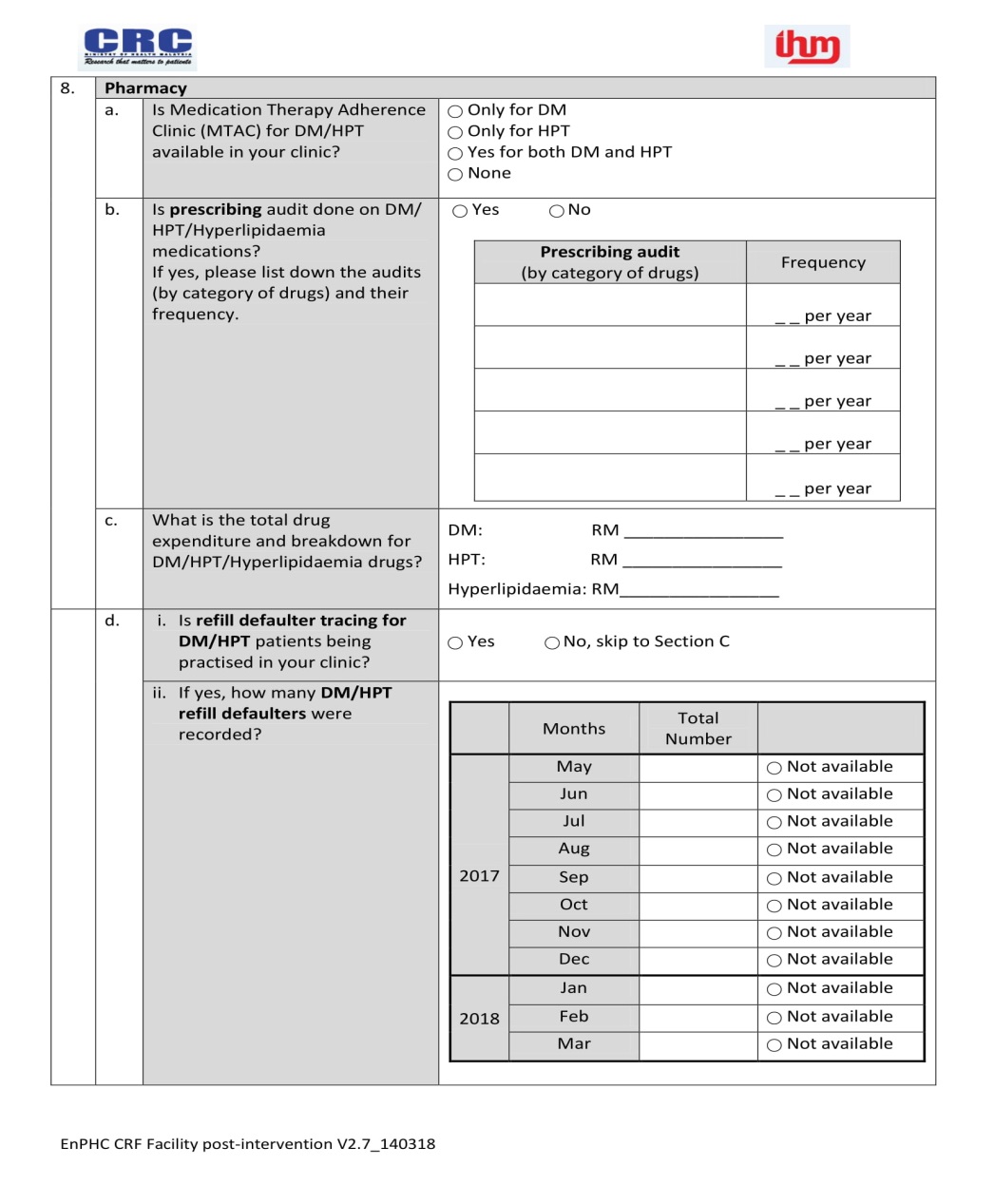

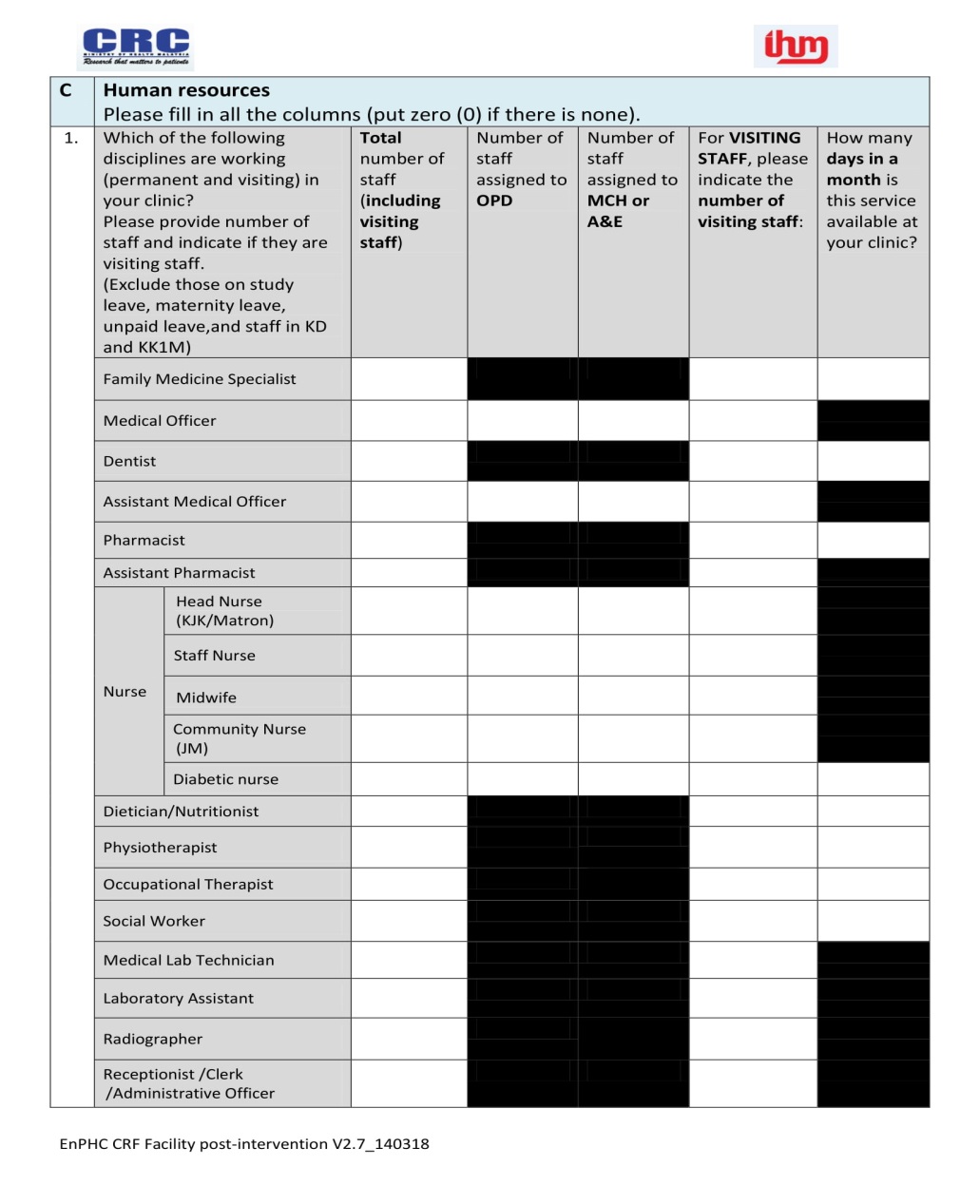


**Appendix D – Facility Questionnaire (Continued)**


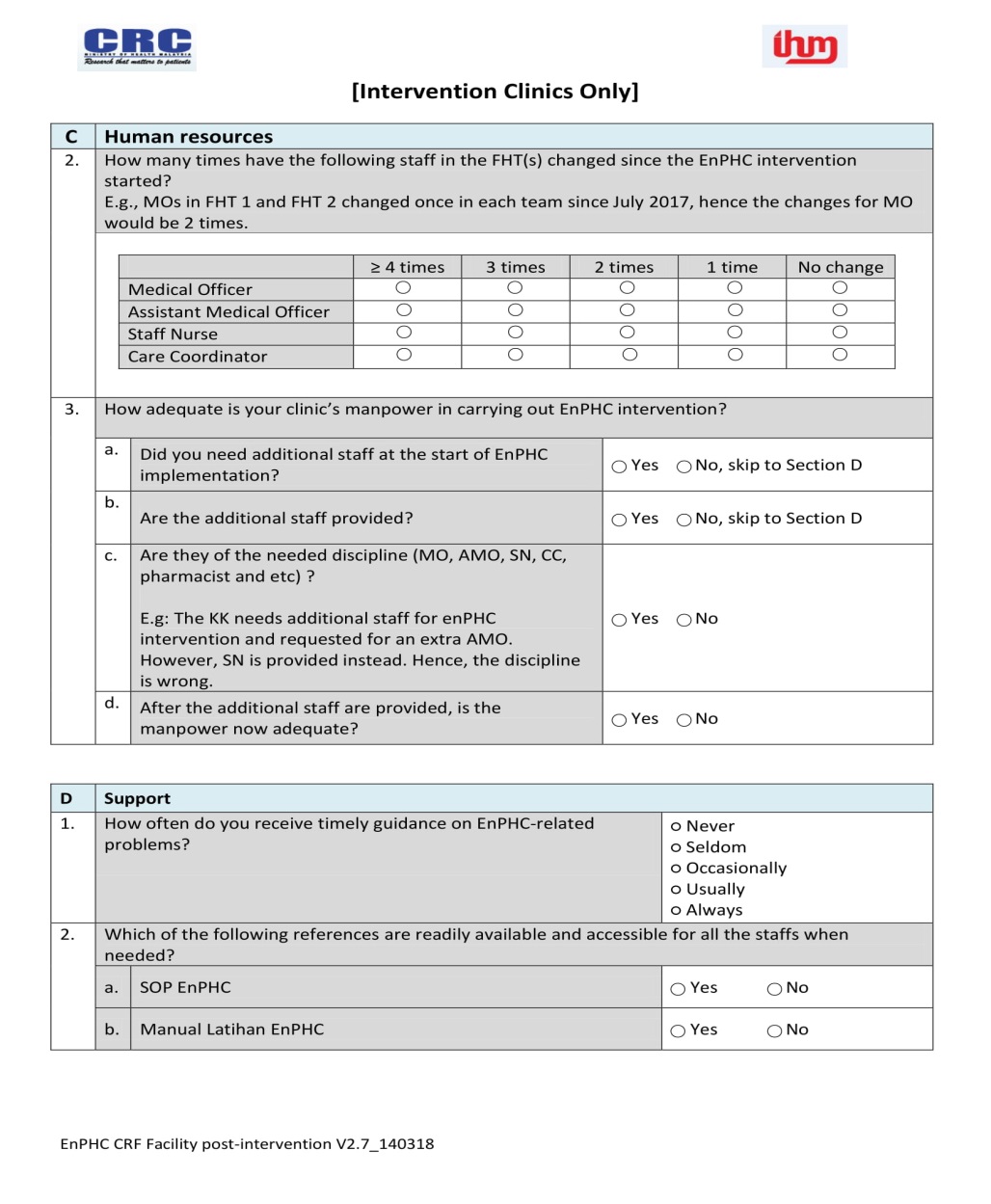

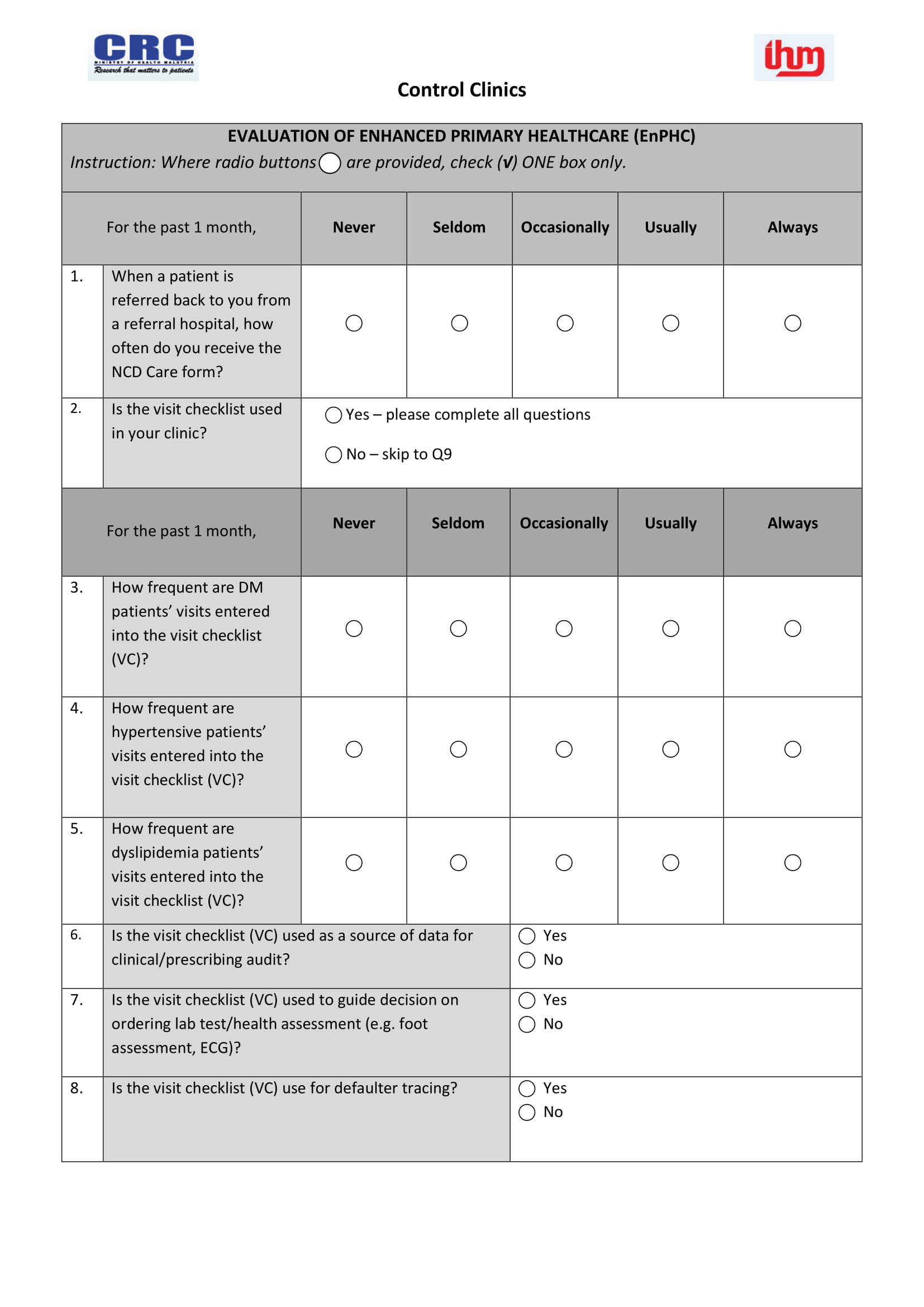


**Appendix D – Facility Questionnaire (Continued)**


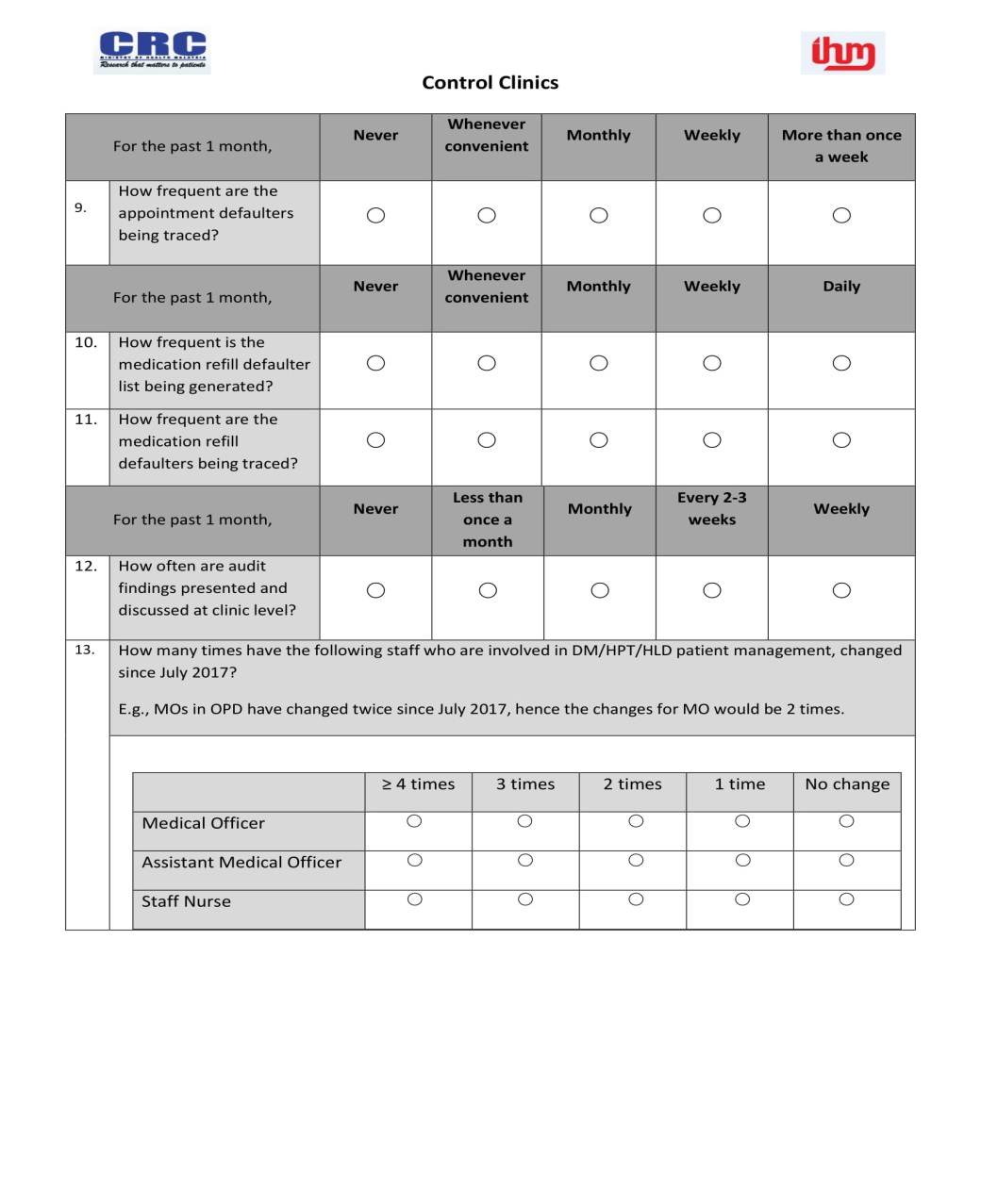


**Appendix E – Intervention Checklist**


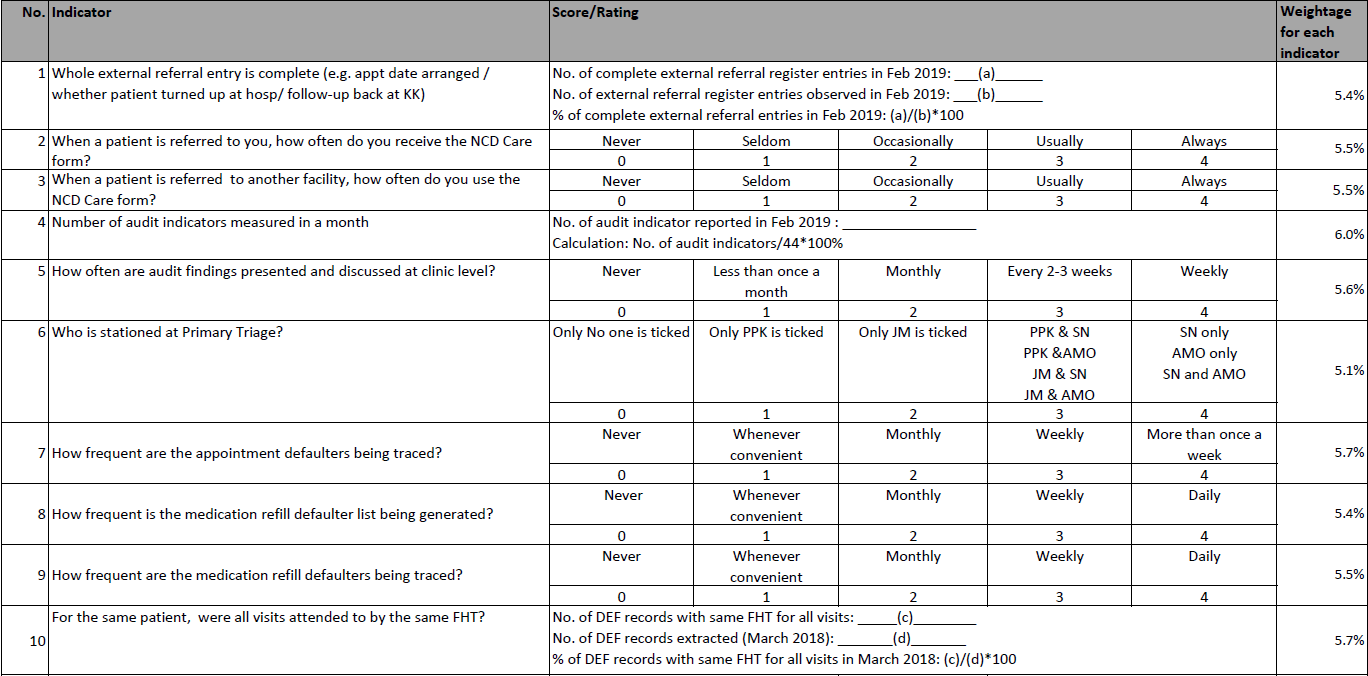


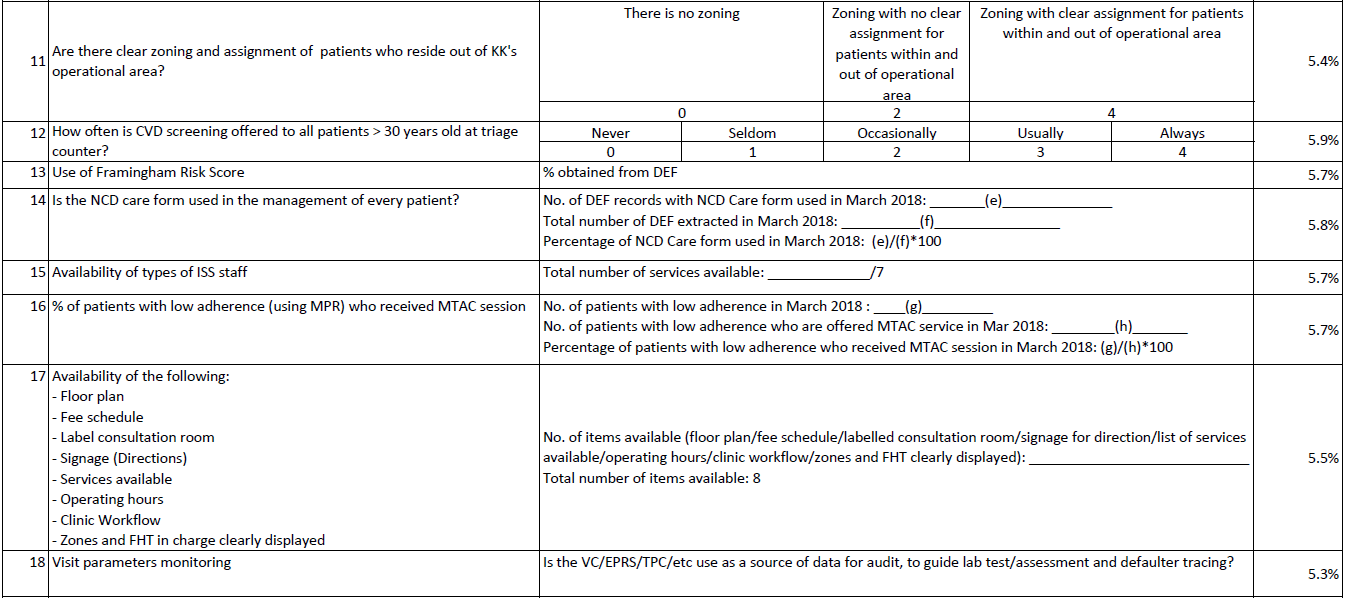


**Appendix F -- Key covariates**

|  | **Processes of Care and Intermediate Clinical Outcomes** | **Patient’s Experience** | **Healthcare Providers Satisfaction** |
| --- | --- | --- | --- |
| ***Patient level*** | Age  Sex  Race  Presence of target organ damage  Morbidities | Age  Sex  Race  Income  Morbidities  Disease duration  General health status  Difference between actual and ideal consultation time | Age  Sex  Education level  Job categories  Practice duration  Practice duration in primary care |
| ***Clinic level*** | Urban/Rural  Degree of Implementation | Urban/Rural  Degree of Implementation | Urban/Rural  Degree of Implementation |
